# Insights into mechanistic interpretation of crystalline-state reddish phosphorescence of non-planar π-conjugated organoboron compounds†

**DOI:** 10.1039/d4sc01184h

**Published:** 2024-04-25

**Authors:** Yohei Adachi, Maho Kurihara, Kohei Yamada, Fuka Arai, Yuto Hattori, Keita Yamana, Riku Kawasaki, Joji Ohshita

**Affiliations:** a Smart Innovation Program, Graduate School of Advanced Science and Engineering, Hiroshima University Higashi-Hiroshima 739-8527 Japan yadachi@hiroshima-u.ac.jp jo@hiroshima-u.ac.jp; b Applied Chemistry Program, Graduate School of Advanced Science and Engineering, Hiroshima University Higashi-Hiroshima 739-8527 Japan; c Division of Materials Model-Based Research, Digital Monozukuri (Manufacturing) Education and Research Center, Hiroshima University Higashi-Hiroshima Hiroshima 739-0046 Japan

## Abstract

Metal-free room-temperature phosphorescent (RTP) materials are attracting attention in such applications as organic light-emitting diodes and bioimaging. However, the chemical structures of RTP materials reported thus far are mostly predominantly based on π-conjugated systems incorporating heavy atoms such as bromine atoms or carbonyl groups, resulting in limited structural diversity. On the other hand, triarylboranes are known for their strong Lewis acidity and deep LUMO energy levels, but few studies have reported on their RTP properties. In this study, we discovered that compounds based on a tetracyclic structure containing boron, referred to as benzo[*d*]dithieno[*b*,*f*]borepins, exhibit strong solid-state reddish phosphorescence even in air. Quantum chemical calculations, including those for model compounds, revealed that the loss of planarity of the tetracyclic structure increases spin–orbit coupling matrix elements, thereby accelerating the intersystem crossing process. Moreover, single-crystal X-ray structural analysis and natural energy decomposition analysis suggested that the borepin compounds without bromine or oxygen atoms, unlike typical RTP materials, exhibit red-shifted phosphorescence in the crystalline state owing to structural relaxation in the T_1_ state. Additionally, the borepin compounds showed potential application as bioimaging dyes.

## Abstract 2

Benzo[*d*]dithieno[*b*,*f*]borepins exhibited reddish room-temperature phosphorescence (RTP) despite the absence of heavy atoms and oxygen atoms.

## Graphical abstract

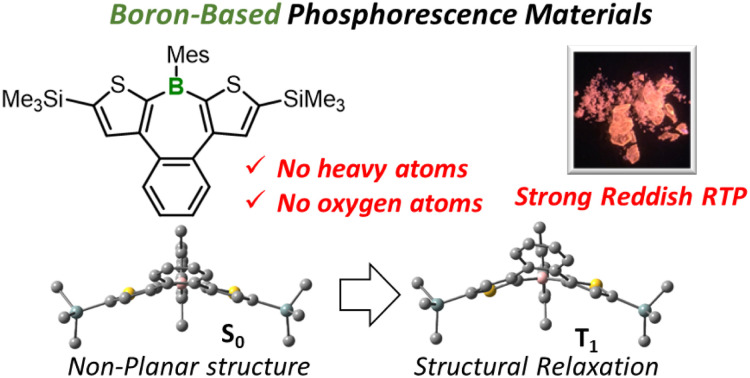

## Introduction

Solid-state phosphorescence has gained prominence in materials chemistry because of its wide-ranging applications such as organic light-emitting diodes, bioimaging, oxygen sensing, and long-persistent luminescent materials.^1^ Phosphorescence is a radiative relaxation process from the triplet excited state to the singlet ground state. Generally, pure organic materials rarely exhibit phosphorescence because of the low rate of intersystem crossing (ISC),^2^ which depends on spin–orbit coupling (SOC). The simplest way to enhance SOC is to introduce heavy metals, such as iridium and platinum, or heavy halogens such as bromine and iodine, which can induce the “heavy atom effect”.^1,3^ As the spin–orbit coupling matrix element (SOCME) of an atom is proportional to the fourth power of its atomic number,^4^ introducing heavy elements dramatically increases the SOCME. However, the use of heavy elements can be disadvantageous in terms of cost, potential toxicity, and the chemical stability of phosphorescent materials. Therefore, there is a substantial demand for the development of heavy element-free organic phosphorescent materials.

**Fig. 1:**
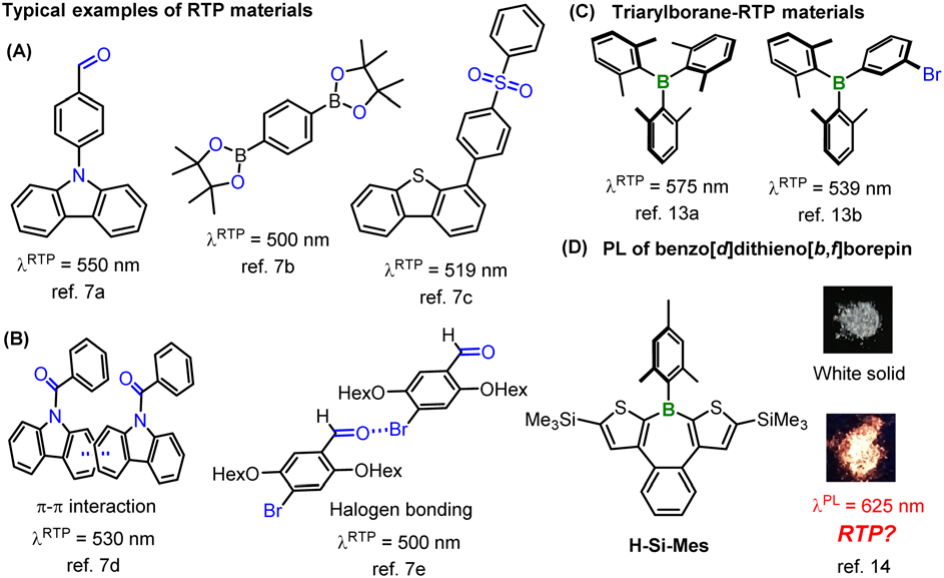
Representative structures of RTP materials (A–C) and borepin compounds (D). The photographs were reprinted with permission from ref. 14. Copyright 2018 American Chemical Society.

Recently, various pure organic room-temperature phosphorescent (RTP) materials have been developed.^5^ In addition to the heavy atom effect, the El-Sayed rule is applied as a common strategy to increase the SOCME.^6^ As a rule, the (π,π*)–(n,π*) transition causes a change in orbital angular momentum that accelerates spin flipping, which is typically observed in conjugated carbonyl compounds such as benzophenone. Various RTP materials synthesized using these strategies, including aromatic amides, carbazoles, aryl sulfones, and aryl boronic acids, among others, have been reported to exhibit solid-state RTP (Fig. 1A and B).^7,8^ Moreover, because of the long phosphorescence emission lifetimes, it is necessary to suppress competing non-radiative decay processes such as internal conversion to achieve strong RTP characteristics. Therefore, it is believed that the induction of strong intermolecular interactions such as π–π interactions or halogen bonding in the solid state can lead to efficient RTP materials (Fig. 1B).^7*d*,*e*,9^ However, these strategies significantly constrain molecular designs for synthesizing new RTP materials. For instance, in reported RTP materials, oxygen or nitrogen atoms bearing an n-orbital are considered essential for leveraging the El-Sayed rule. Moreover, RTP materials showing low-energy RTP in the red or NIR wavelength region are quite rare owing to the fast non-radiative deactivation of low-energy excited states (energy gap law).^10^ In large π-conjugated systems, the transitions to both S_1_ and T_1_ states are predominantly π–π* transitions, thereby rendering the El-Sayed rule ineffective. Consequently, many reported RTP materials are confined to small π-conjugated systems, resulting in greenish to yellowish phosphorescence in most heavy-atom-free RTP systems. A further constraint is that common RTP materials suppress structural relaxation owing to strong intermolecular interactions, resulting in small Stokes shifts. Therefore, heavy-atom-free materials showing reddish RTP typically exhibit absorption in the visible region.^11^ The present strategies for RTP materials synthesis considerably restrict the absorption and RTP wavelengths of compounds, making it challenging to design heavy-atom-free molecules that simultaneously exhibit no absorption in the visible region and reddish RTP, for example.

On the other hand, the introduction of tricoordinate boron into π-conjugated systems is widely known to significantly alter the optical properties of π-conjugated materials because of the orbital interaction between boron's empty 2p orbital and π* orbitals (p–π* interaction). The high Lewis acidity and the low-lying LUMO energy levels of triarylboranes make these compounds suitable for use as Lewis acid catalysts, anion sensor materials, and n-type semiconductors.^12^ However, as boron has no n orbital, there are few examples of RTP materials based on triarylboranes (Fig. 1C).^13^ Previously, we discovered that a benzo[*d*]dithieno[*b*,*f*]borepin compound (H-Si-Mes, Fig. 1D) exhibits reddish photoluminescence (PL) in the crystalline state.^14^ However, we were unable to elucidate the detailed mechanism underlying this PL. Of the benzo[*d*]dithieno[*b*,*f*]borepin-based polymers and fluorescent materials that we synthesized, none exhibited the aforementioned reddish PL in the solid state.^15^ In this study, aiming to re-examine the mechanism of this reddish PL, we synthesized borepin compounds with different substituents on the boron atom or the aromatic rings, in addition to H-Si-Mes, and investigated their PL properties in the crystalline state. Surprisingly, several borepin compounds including H-Si-Mes exhibited reddish PL in the crystalline state despite only absorbing in the UV region, leading to a large Stokes shift. In addition, it was revealed that the reddish PL is phosphorescence (RTP). Furthermore, by isolating crystals that showed RTP from those that did not, and analyzing them by single-crystal X-ray diffraction and DFT calculations, we revealed that unlike common RTP materials, structural relaxation in the crystal may enhance the RTP properties of these borepin compounds. The chemical structures of these borepin compounds exhibiting such unique RTP properties diverge markedly from those of reported RTP materials, suggesting they belong to a new class of RTP materials. We also explored the application of this unique RTP property by investigating the utility of H-Si-Mes as a bioimaging dye.

## Results and discussion

### Synthesis

**Scheme 1:**
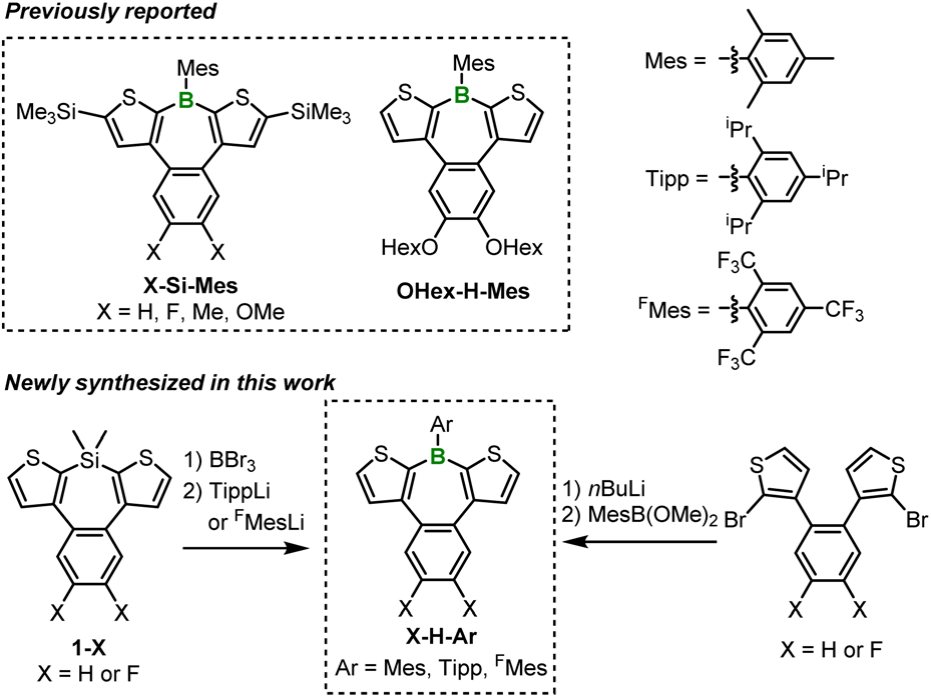
Chemical structures of benzo[*d*]dithieno[*b*,*f*]borepins reported previously and synthesized in this work.

The borepin derivatives synthesized in this study were named as follows. As mentioned earlier, benzo[*d*]dithieno[*b*,*f*]borepins allow for the introduction of substituents on the boron atom, the benzene ring, and the thiophene rings. In this study, we introduced bulky aryl substituents on boron, such as mesityl (Mes), 2,4,6-triisopropylphenyl (Tipp), and 2,4,6-tris(trifluoromethyl)phenyl (^F^Mes) groups.^16^ The α-positions of the thiophene rings were either left unsubstituted or substituted with trimethylsilyl (TMS) groups. In addition, hydrogen (not substituted), fluorine, or hexyloxy groups were introduced on the benzene ring. On the basis of these structural variations, the compounds were assigned names in the order of substitutions on the benzene ring, substitutions on the thiophene rings, and aryl substitution on boron. For example, if there are no substitutions on the benzene ring, TMS substitutions on the thiophene rings, and Mes substitution on boron, the compound is named H-Si-Mes. Compounds with Mes-substituted boron, such as X-Si-Mes (X = H, F, Me, OMe)^14^ and OHex-H-Mes,^15*b*^ have been reported in previous studies (Scheme 1). Among the X-Si-Mes series, however, only H-Si-Mes exhibited reddish PL.^14^ Accordingly, in this study, we focused our analysis exclusively on H-Si-Mes in this series.

In addition to the abovementioned compounds, we newly synthesized a total of six compounds, denoted as X-H-Ar, having a Mes, Tipp, or ^F^Mes group as the boron substituent and either hydrogen or fluorine atoms as benzene ring substituents (Scheme 1). X-H-Tipp and X-H-^F^Mes were prepared *via* a B–Si exchange reaction with corresponding silepins 1-X, followed by a nucleophilic substitution reaction with aryllithium. X-H-Mes were synthesized using a similar method to the preparation of H-Si-Mes.^14^ The substituents and aryl group introduced into the benzo[*d*]dithieno[*b*,*f*]borepin structure do not significantly extend conjugation. Therefore, in diluted solutions, the photophysical properties of X-H-Ar are expected to be similar to that of H-Si-Mes, which previously exhibited reddish PL in the solid state. However, if these compounds demonstrated different PL properties (*e.g.*, blueish or reddish emission) from each other in the crystalline state, their PL spectra and single-crystal X-ray analysis would reveal factors contributing to the expression of the reddish PL properties. Indeed, as described below, we obtained intriguing crystal polymorphs for three compounds, X-Si-Mes, OHex-H-Mes, and F-H-^F^Mes, which displayed distinguishable PL properties in the solid state.

### Optical properties in solution

**Fig. 2:**
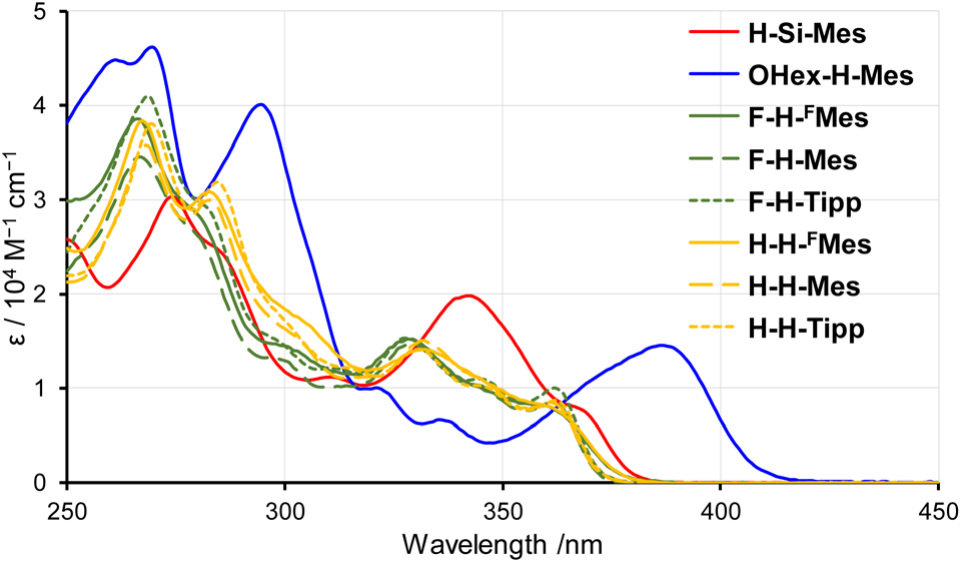
UV-vis absorption spectra of benzo[*d*]dithieno[*b*,*f*]borepins in THF.

To investigate the influence of substituents on the electronic states of borepin compounds, we measured their UV-vis absorption and PL spectra in THF. The UV-vis absorption spectra of X-H-Ar compounds showed a remarkable resemblance to each other (Fig. 2 and Table 1), suggesting that the electronic effects of fluorine atoms on the benzene ring and aryl substituents on the boron atom have a relatively limited impact on the tetracyclic borepin structure. The comparison between the spectra of H-H-Mes and H-Si-Mes revealed that the introduction of TMS groups caused a slight red shift in the absorption edge, indicating a minor influence of the TMS groups on the electronic structure of the borepin core. The absorption band of OHex-H-Mes was notably red-shifted relative to that of H-H-Mes, possibly because of the intramolecular donor–acceptor interactions between the electron-donating alkoxy group and the electron-deficient borepin unit. Nonetheless, the absorption bands of all the borepin compounds were observed in the UV region, suggesting that the substituents had little impact on the electronic structures of benzo[*d*]dithieno[*b*,*f*]borepins. All the compounds have molar absorption coefficients of over 6500 at 365 nm, suggesting that they can be photoexcited by common UV light sources.

**Table 1:** Optical properties of benzo[*d*]dithieno[*b*,*f*]borepins in THF

Compound	*λ* ^Abs^_onset_ a/nm	*λ* ^PL^ b/nm	*Φ*_PL_ c	*τ* ^FL^ d/ns	*λ* ^Phos^ e/nm	*τ* ^Phos^ f/ms
H-Si-Mes	381	383	<0.02	0.25	493	161
OHex-H-Mes	408	429	<0.02	—g	492	166
F-H-^F^Mes	377	382	<0.02	0.29	486	155
F-H-Mes	373	374	<0.02	0.22	—g	—g
F-H-Tipp	374	374	<0.02	0.26	—g	—g
H-H-^F^Mes	377	385	<0.02	0.31	—g	—g
H-H-Mes	373	374	<0.02	0.25	—g	—g
H-H-Tipp	374	373	<0.02	0.31	—g	—g

a Absorption onset.

b Fluorescence maximum.

c Absolute PL quantum yield at room temperature.

d Fluorescence lifetime at room temperature.

e Phosphorescence maximum in 2-MeTHF at 77 K.

f Phosphorescence lifetime in 2-MeTHF at 77 K.

g Not measured.

**Fig. 3:**
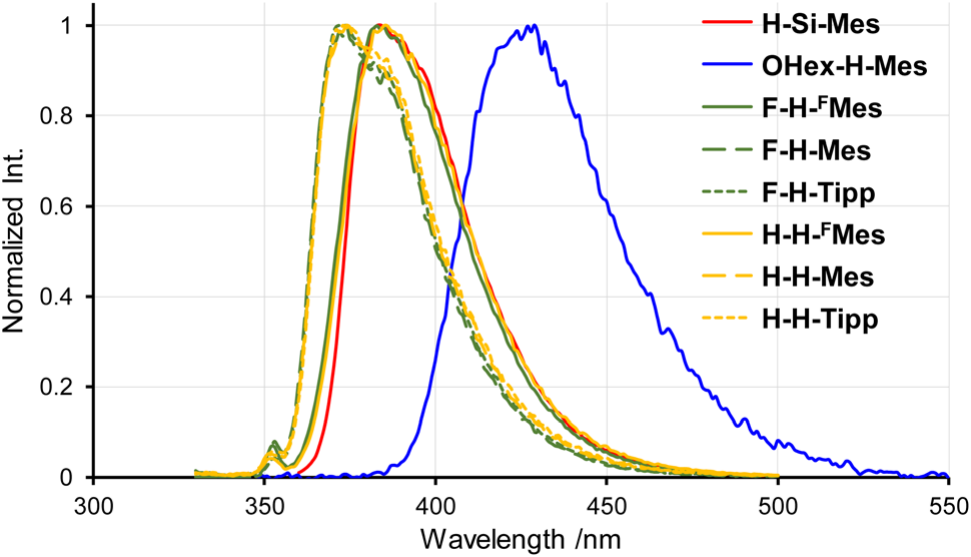
PL spectra of benzo[*d*]dithieno[*b*,*f*]borepins in THF at room temperature.

**Fig. 4:**
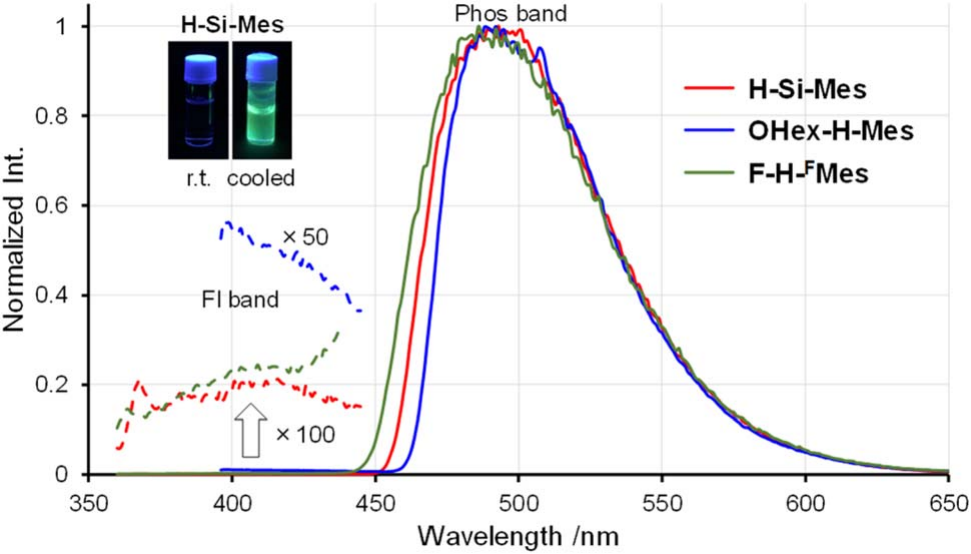
Phosphorescence spectra of benzo[*d*]dithieno[*b*,*f*]borepins in 2-MeTHF at 77 K.

The PL spectra in solution displayed a similar trend to that observed in the UV-vis absorption spectra (Fig. 3). Only the emission band of OHex-H-Mes was slightly red-shifted compared with the other borepin compounds. The emission maxima were in the UV region around 380 nm for most borepin compounds, suggesting that the influence of the introduced substituents on the excited state is also limited in solution. The lifetimes of these emissions were very short (Table 1 and Fig. S1†). The emission intensity remained unchanged when argon bubbling was performed before measurements (Fig. S2†). These results clearly indicate that they are usual fluorescence. Importantly, the PL quantum yields (*Φ*_PL_) of all the borepin compounds in THF were less than 2% (Table 1). The deactivation process of the photoexcited singlet state occurred because of internal conversion either from the lowest singlet excited state (S_1_) or from the triplet state generated *via* ISC. To confirm the involvement of ISC processes in the deactivation process, we measured phosphorescence spectra at 77 K in a 2-MeTHF glass matrix. The obtained phosphorescence spectra are shown in Fig. 4. In contrast to the very weak fluorescence at room temperature, at 77 K, strong phosphorescence and distinct greenish PL were observed. Such low-temperature phosphorescence has also been reported for other triarylborane compounds.^17^ The lifetime of this greenish PL was on the order of milliseconds (155–166 ms, Table 1 and Fig. S3†), clearly indicating that the observed PL is phosphorescence. These findings suggest that the non-radiative deactivation process of the borepin compounds at room temperature is predominantly governed by internal conversion from the triplet state generated *via* ISC from the excited singlet states.

### Crystals of benzo[*d*]dithieno[*b*,*f*]borepins and their solid-state PL properties

**Fig. 5:**
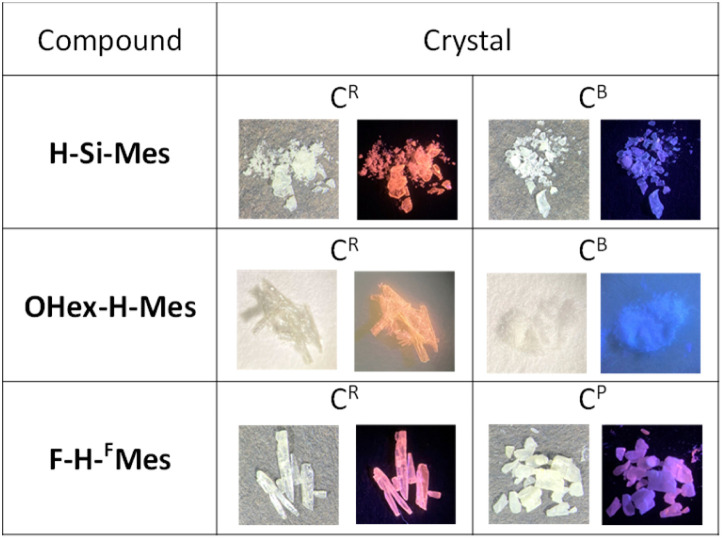
Photographs of recrystallized crystals of benzo[*d*]dithieno[*b*,*f*]borepins taken under room light or UV (365 nm).

We have previously reported that H-Si-Mes crystals exhibit strong reddish PL.^14^ To re-examine this property, H-Si-Mes was recrystallized from hexane solution, resulting in the formation of crystals that emit reddish PL (H-Si-Mes_C^R^) as previously reported, as well as crystals that emit blueish PL (H-Si-Mes_C^B^) (Fig. 5). H-Si-Mes_C^R^ and H-Si-Mes_C^B^ were not formed simultaneously; either one of the crystals was selectively obtained upon each recrystallization attempt. Unfortunately, despite attempting various recrystallization solvents and temperatures, we were unable to reproducibly control the formation of either the C^R^ or C^B^ crystals. OHex-H-Mes and F-H-^F^Mes also exhibited a similar crystalline polymorphism to that of H-Si-Mes (Fig. 5). Recrystallization of OHex-H-Mes and F-H-^F^Mes from hexane solutions yielded crystals emitting blueish PL (OHex-H-Mes_C^B^) and crystals emitting pinkish PL (F-H-^F^Mes_C^P^) in addition to crystals emitting reddish PL (C^R^ crystals), respectively. However, unlike H-Si-Mes, those crystals of OHex-H-Mes and F-H-^F^Mes formed simultaneously in the same vessel. Therefore, those crystals were manually separated after recrystallization. Photographs of the crystals of these three compounds obtained from recrystallization are shown in Fig. 5. All the crystals were colorless or off-white, which indicated minimal absorption in the visible region. Except for the three compounds above, no crystalline polymorphism was exhibited for other borepins. As shown in Fig. S4,† the crystals of X-H-Ar also exhibited varied emission colors ranging from blueish to reddish. In addition, the amorphous samples of X-H-Ar prepared by melting and rapid cooling did not exhibit reddish PL, consistent with the trend previously reported for H-Si-Mes.^14^

**Fig. 6:**
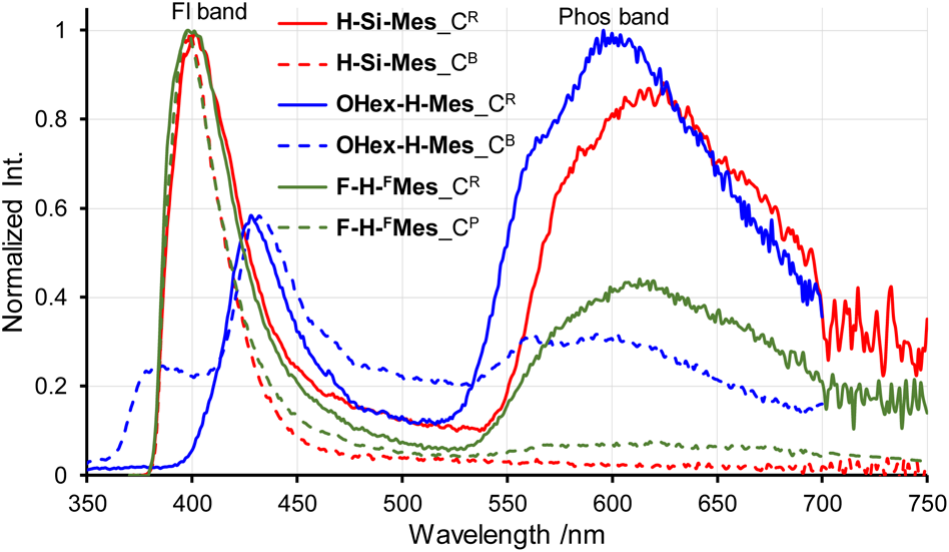
PL spectra of crystals of benzo[*d*]dithieno[*b*,*f*]borepins with crystal polymorphism in air at room temperature.

Next, the PL spectra of H-Si-Mes crystals were measured. In the case of C^R^ crystals, in addition to the PL band around 400 nm, a band around 600 nm was observed (Fig. 6 and Table 2). The observed dual PL bands matched the PL bands previously reported for H-Si-Mes crystals.^14^ The PL quantum yield (*Φ*_PL_) of H-Si-Mes_C^R^ obtained in the present study was 12%, which was roughly the same as the previously reported *Φ*_PL_ of 18%,^14^ suggesting that the structures of H-Si-Mes_C^R^ obtained in the present and previous studies are the same. The PL lifetime of H-Si-Mes_C^R^ was measured, revealing a short-wavelength PL band with a lifetime of 0.17 ns (*τ*^FL^ in Table 2 and Fig. S5†), like that observed in solution at room temperature, indicating that it is fluorescence. In contrast, the long-wavelength PL band around 600 nm had a very long phosphorescence lifetime of 21.3 ms (*τ*^Phos^ in Table 2 and Fig. S6†), which indicates that this reddish PL is RTP. As there are very few examples of triarylboranes without heavy atoms or oxygen atoms exhibiting RTP properties,^13*a*^ it is of high interest that the present borepin compounds exhibit reddish RTP. In contrast to H-Si-Mes_C^R^, no long-wavelength phosphorescence was observed in the PL spectrum of H-Si-Mes_C^B^ (Fig. 6), suggesting that the RTP properties are dependent on the crystal structures. This was also the case for OHex-H-Mes and F-H-^F^Mes, with their respective C^R^ crystals exhibiting strong phosphorescence; however, the intensity of the phosphorescence band decreased in the C^B^ and C^P^ crystals (Fig. 6), as evident from the intensity ratio of phosphorescence and fluorescence bands (*I*^Phos^/*I*^Fl^ in Table 2). However, the crystalline state did not significantly affect the PL wavelengths. In the excitation spectra of the C^R^ crystals (Fig. S7†), the bands were observed only in the UV region, which is consistent with the crystals being colorless. The excitation bands of the C^R^ crystals were not significantly different from the UV absorption in solution (Fig. 2), suggesting the absence of strong intermolecular interactions even in the crystalline state. Since phosphorescence is typically quenched by oxygen, the PL of H-Si-Mes_C^R^ in air and vacuum were compared, however, no significant difference was observed (Fig. S8†). This may be due to the slow diffusion of oxygen into the crystals.

**Table 2:** Optical properties of benzo[*d*]dithieno[*b*,*f*]borepins in the solid state

Compound	Crystal	*λ* ^FL^ a/nm	*λ* ^Phos^ b/nm	*Φ*_PL_ c	*I* ^Phos^/*I*^Fl^^d^	*τ* ^FL^ e/ns	*τ* ^Phos^ f/ms
H-Si-Mes	C^R^	402	625	0.12	0.89	0.17 (100)	21.3
	C^B^	399	—g	<0.02	<0.02	0.17 (100)	—g
OHex-H-Mes	C^R^	428	596	0.09	1.71	0.13 (94), 3.01 (6)	9.87
	C^B^	432	593	<0.02	0.54	0.18 (98), 1.20 (2)	—g
F-H-^F^Mes	C^R^	398	613	0.03	0.44	0.20 (98), 2.50 (2)	5.36
	C^P^	398	618	<0.02	0.08	0.07 (83), 0.24 (17)	—g

a Fluorescence maximum.

b Phosphorescence maximum.

c Absolute PL quantum yield including both fluorescence and phosphorescence bands at room temperature.

d Intensity ratio of fluorescence and phosphorescence bands.

e Fluorescence lifetime with percentage lifetime contribution in parentheses.

f Phosphorescence lifetime.

g Not measured.

Different from the above three compounds, other borepin compounds showed no polymorphism. The solid-state PL spectra of X-H-Ar, except F-H-^F^Mes, revealed phosphorescence bands of varying intensities relative to the fluorescence bands (Fig. S4, S9 and Table S1†), similar to those showing polymorphism. However, no correlation was observed between the intensities of the phosphorescence bands and the chemical structures, including the aryl substituents and the benzene ring substituents. This indicated that the RTP properties of benzo[*d*]dithieno[*b*,*f*]borepins were not solely due to the presence of specific substituents. The borepin compounds exhibited three notable characteristics related to their RTP properties: (1) rapid ISC to the triplet states despite the absence of heavy atoms or oxygen atoms; (2) significantly different phosphorescence wavelengths observed in glassy matrices at 77 K compared with those observed in the crystalline state; and (3) crystal structure-dependent RTP properties (as observed in C^R^ and C^B^/C^P^ crystals). To gain an insight into the molecular design necessary for developing metal-free RTP materials, quantum chemical calculations and single-crystal X-ray structural analysis were performed to further elucidate these features.

### DFT calculations

**Fig. 7:**
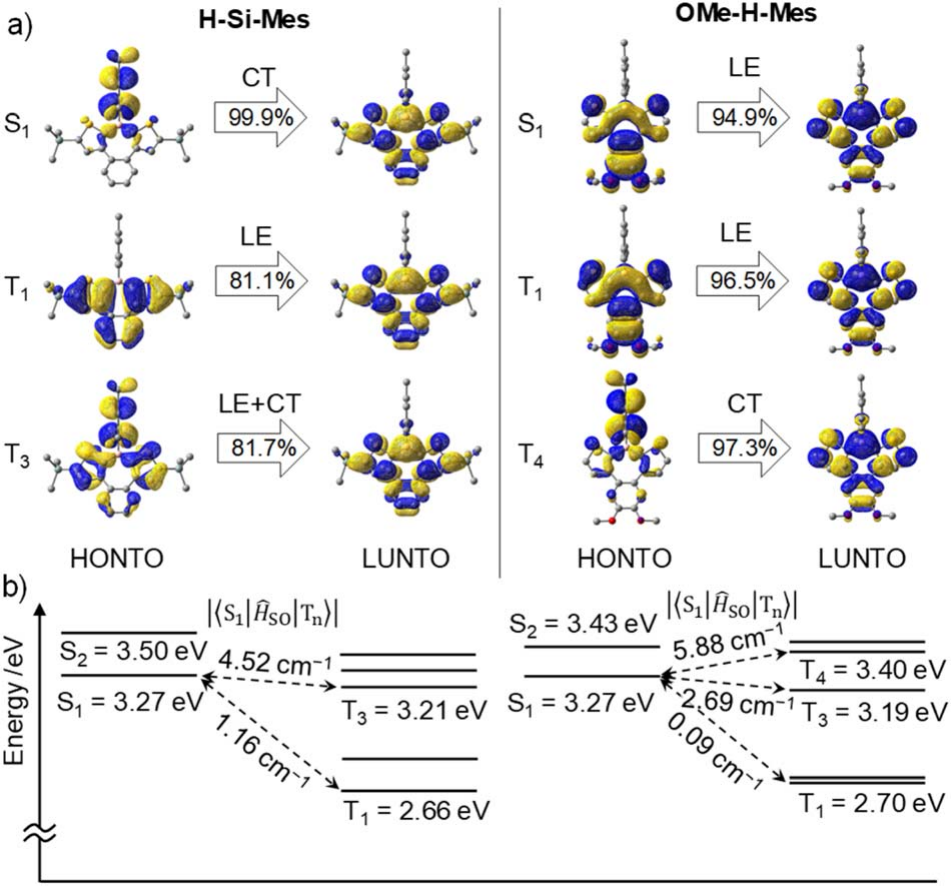
(a) NTOs of H-Si-Mes and OMe-H-Mes in the S_0_ geometry at the B3LYP/6-31G(d) level of theory. (b) Calculated singlet and triplet energy levels and SOCMEs at the B3LYP/6-31G(d) level of theory.

First, we performed geometry optimization for H-Si-Mes in the S_0_ state in the gas phase at the B3LYP/6-31G(d) level. The *C*_s_ symmetric optimized structure had low planarity, which is due to the steric repulsion between the benzene and thiophene rings (Fig. S10†). The optimized structure generally matched the single-crystal X-ray structure of H-Si-Mes_C^R^.^14^ The optimized structures of F-H-^F^Mes and OMe-H-Mes, in which the hexyl groups of OHex-H-Mes are substituted with methyl groups to reduce computational cost, were non-planar, similar to that of H-Si-Mes (Fig. S10†). Next, TD-DFT calculations were performed on the optimized S_0_ structure, and the transitions were visualized through natural transition orbital (NTO) analysis (Fig. 7 and S11–S13†). The S_1_ state of H-Si-Mes corresponded to a transition from HOMO to LUMO, with a small oscillator strength of 0.004 (Table S2†). The NTO analysis revealed that the hole localized on the Mes group that was introduced as a bulky substituent for kinetic stabilization, and the particle delocalized on the tetracyclic structure with the p-orbital of boron (Fig. 7). This indicates that the S_1_ state of H-Si-Mes has a twisted charge-transfer (CT) character. On the other hand, the transitions from T_1_ to T_4_ mainly were local excitations (LE) within the tetracyclic structure containing the borepin ring. These results suggest the potential for efficient generation of triplet states in H-Si-Mes through spin–orbit charge transfer intersystem crossing (SOCT-ISC).^18^ In addition, a structure–property relationship was developed computationally and verified experimentally in solution, PMMA films, and in OLED devices, whereby tuning of the ISC and reverse ISC processes in donor–acceptor triarylboranes can be achieved by adjusting the energy of the local excited state (^3^LE_π_) *via* modification of the bridging group between the donor and tricoordinate boron acceptor.^19^ ISC is accelerated when energy differences between the S_1_ and triplet states are small and when SOCMEs are large. For H-Si-Mes, SOCMEs between the S_1_ and triplet (T_1_–T_5_) states were calculated to be in the range of 1.16 to 5.55 cm^−1^, and these values are sufficiently large for ISC to occur (Fig. 7 and Table S3†). This contrasts with the very small SOCMEs of 0.00 to 0.16 between the S_1_ and T_*n*_ states in naphthodithiophene 2 without boron bridging (Table S3,†*vide infra*). Relatively large SOCMEs, as in the case of H-Si-Mes, were also obtained for OMe-H-Mes and F-H-^F^Mes (Table S3†). To investigate the influence of structural relaxation in the S_1_ state on SOCME, the S_1_ structures of these three compounds were optimized using TD-DFT, and SOCMEs were calculated for the optimized S_1_ structures. While the planarity slightly decreased in the S_1_ structures compared to the corresponding S_0_ structures, *C*_s_ symmetries were maintained, and significant structural relaxation did not occur (Fig. S10†). Therefore, the values of SOCMEs in the S_1_ structure did not differ significantly from those in their S_0_ structures (Table S4†).

**Fig. 8:**
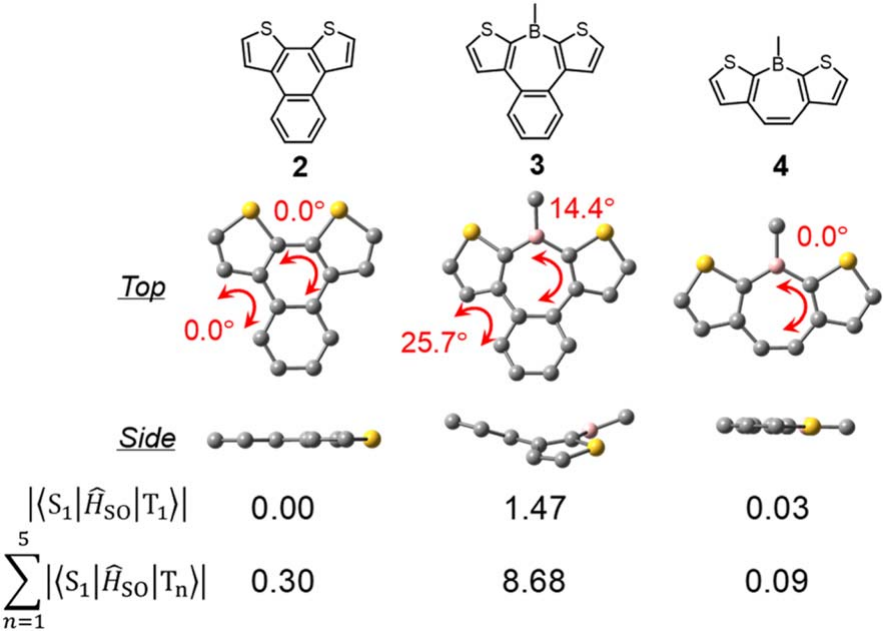
DFT-optimized S_0_ geometries of model compounds and their SOCMEs in cm^−1^.

To elucidate the effects of the borepin ring and the aryl groups on boron in the ISC process from the S_1_ state to the triplet states, TD-DFT and SOCME calculations were performed on model compounds including H-H-Mes, benzo[*d*]dithieno[*b*,*f*]silepin 1-H, naphthodithiophene 2, benzo[*d*]dithieno[*b*,*f*]borepin 3 with a methyl group on boron, and dithieno[*b*,*f*]borepins 4 and 5 with a methyl group and a mesityl group on boron, respectively (Fig. 8 and S14†). The TD-DFT calculation results are summarized in Table S5.† Initially, geometry optimizations in the ground state revealed that compounds 2, 4, and 5 have a planar tri- or tetracyclic π-conjugated system, whereas H-H-Mes, 1-H, and 3 exhibit deviations from planarity owing to steric repulsion between the benzene and thiophene rings, as in H-Si-Mes (Fig. 8 and S14†). Comparisons of SOCMEs in these model compounds clearly showed that compounds with lower planarity have significantly higher SOCMEs (Fig. 8 and Table S3†). For example, SOCMEs between the S_1_ and T_1_ states were only 0.00 and 0.03 cm^−1^ in highly planar compounds 2 and 4, respectively, whereas that in 3, a compound with reduced planarity, was much larger at 1.47 cm^−1^. The ISC from the S_1_ state may occur not only to the T_1_ state but also to the energetically proximate T_*n*_ states. In triarylboranes exhibiting RTP, it has been reported that ISC from the S_1_ state to higher triplet states such as T_2_ is more efficient than from S_1_ to T_1_.^13*a*,*b*^ In the case of benzo[*d*]dithieno[*b*,*f*]borepins that show the large energy difference between S_1_ and T_1_ states, it is also likely that ISC from S_1_ to higher triplet states like T_3_ or T_4_ is involved. Therefore, the sum of SOCMEs from S_1_ to T_*n*_ (*n* = 1–5) was calculated and compared.^20^ The sum of SOCMEs of compounds 2, 3, and 4 was calculated to be 0.30, 8.68, and 0.09 cm^−1^, respectively, showing significant differences (Fig. 8). In the case of polyaromatic compounds such as perylene imide, twisting of the π-conjugated framework has been reported to greatly accelerate ISC.^21^ Therefore, in benzo[*d*]dithieno[*b*,*f*]borepins, the non-planar structure of the tetracyclic π-conjugated system may also contribute to the promotion of ISC. Indeed, highly planar compound 5 has been found to exhibit fluorescence at room temperature (*Φ*_PL_ = 0.05).^22^ In the present study, the *Φ*_PL_ values of non-planar borepins (*e.g.*, H-Si-Mes) were even lower (*Φ*_PL_ < 0.02, Table 1). To further investigate the importance of non-planar structures, the optical properties of silepin 1-H were examined. Like H-Si-Mes, silepin 1-H having a non-planar structure had large SOCMEs (Table S3†) and exhibited very low absolute fluorescence quantum yield at room temperature (*Φ*_PL_ < 0.02, Fig. S15†). This contrasts with the moderately high quantum yield observed for planar 5,5-dimethyldibenzo[*b*,*f*]silepin (*Φ*_PL_ = 0.14 in CH_2_Cl_2_).^23^ Furthermore, compound 1-H exhibited stronger phosphorescence than H-Si-Mes at 77 K (Fig. S16†). These results strongly support the tendency for non-planar structures to enhance ISC efficiency. Additionally, there is a possibility that the thiophene rings facilitate ISC. Compounds combining tricoordinate boron with EDOT have been reported to efficiently generate triplet excited states.^24^

Next, the energy gap between S_1_ and T_1_ (Δ*E*_ST_) was compared among the model compounds. Δ*E*_ST_ values were relatively large, exceeding 0.6 eV in all cases (Table S3†), suggesting that direct ISC from S_1_ to T_1_ may not be very effective. However, Δ*E*_ST_ is still an important parameter when discussing how each molecular structure affects the energies of the singlet and triplet states. Compared with simple aromatic hydrocarbon 2 (Δ*E*_ST_ = 1.33 eV), compounds with boron exhibited a significant reduction in Δ*E*_ST_ (*e.g.*, H-H-Mes: Δ*E*_ST_ = 0.63 eV). This suggests that the borepin structure contributes to the decrease in Δ*E*_ST_. Further comparisons between compound 3 and H-H-Mes or compounds 4 and 5 revealed that changing the substituents on boron from the methyl group to the mesityl group led to a decrease in Δ*E*_ST_ and a significant improvement in SOCMEs, possibly because of the involvement of the aforementioned CT transitions. These results indicate that in benzo[*d*]dithieno[*b*,*f*]borepins including H-Si-Mes, the non-planar borepin structures and the aryl groups on boron synergistically enhance SOCMEs and reduce Δ*E*_ST_.

**Fig. 9:**
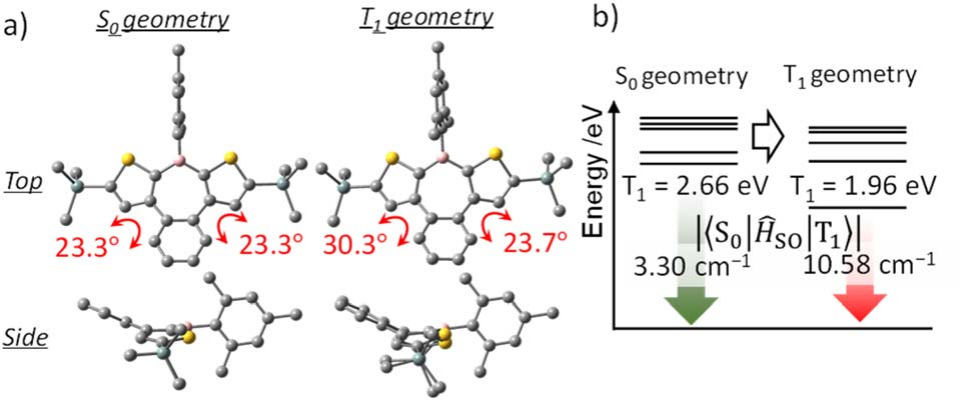
Optimized structures (a), triplet energy levels, and SOCMEs (b) of H-Si-Mes in S_0_ and T_1_ geometries at the B3LYP/6-31G(d) level of theory.

Because phosphorescence is a relaxation process from T_1_ to S_0_, structural relaxation in the T_1_ state may affect the emission wavelength and the ISC efficiency. Accordingly, the influence of structural relaxation in the T_1_ state of benzo[*d*]dithieno[*b*,*f*]borepins was investigated. Optimization of the T_1_ state of H-Si-Mes using TD-DFT yielded a slightly less planar structure than the S_0_ state (Fig. 9a and S10†). In the T_1_ structure, the borepin ring was asymmetrically bent, and the symmetry decreased to *C*_1_. The T_1_–S_0_ transition energy, which was calculated to be 2.66 eV (466 nm) in the S_0_ structure, significantly decreased to 1.96 eV (633 nm) in the optimized T_1_ structure (Fig. 9b and Table S6†). This small transition energy roughly matched the observed reddish phosphorescence in the spectrum of H-Si-Mes_C^R^, suggesting that the structural relaxation in the T_1_ state has a significant impact on the phosphorescence wavelength. Similar calculation results were obtained for OMe-H-Mes and F-H-^F^Mes (Tables S6†). At 77 K in a glass matrix, higher-energy green phosphorescence was observed, which is likely attributable to the suppression of structural relaxation at low temperatures, thereby allowing phosphorescence from the higher-energy quasi-stable T_1_ state to occur. The SOCMEs between the S_0_ and T_1_ states calculated for H-Si-Mes to compare ISC efficiency in the phosphorescence process were 3.30 and 10.58 cm^−1^ in the S_0_ and T_1_ structures, respectively (Fig. 9, Tables S3 and S7†). The increased S_0_–T_1_ SOCME in the T_1_ structure potentially enhanced the RTP properties observed in the C^R^ crystals. When the variable-temperature (VT) PL spectra of H-Si-Mes_C^R^ (Fig. S17†) were measured, the intensity of the long-wavelength phosphorescence band decreased with increasing measurement temperature, which is typical behavior for phosphorescence. Interestingly, a new emission band appeared around 520 nm at low temperatures. This new emission band had a long lifetime contribution of 182 ms (Table S8†) and was thus confirmed to be phosphorescence. The wavelength and lifetime of this new phosphorescence band closely matched those of the phosphorescence band observed in the glass matrix at 77 K, supporting the presence of the quasi-stable T_1_ state described above. The increase in SOCME from S_0_ to T_1_ was observed not only in H-Si-Mes, which exhibited prominent RTP characteristics in the C^R^ crystals, but also in H-H-Mes, which showed inferior RTP characteristics (Fig. S9, Tables S3 and S7†). This clearly suggests that the effect of the substituents on ISC efficiency as a monomeric unit of benzo[*d*]dithieno[*b*,*f*]borepins is not significant, and that the packing structure of the crystal has a significant impact on the RTP characteristics in the solid state.

### Crystal structures

Next, we investigated the influence of crystal structures on RTP properties. Fortunately, we were able to obtain a total of six single-crystal X-ray structures for two types of crystals with differing RTP properties for each of the three compounds (H-Si-Mes,^14^F-H-^F^Mes, and OHex-H-Mes, CCDC 2324621–2324625†). In the case of H-Si-Mes, neither C^R^ nor C^B^ crystals contained solvent molecules (Fig. S18†). However, in F-H-^F^Mes_C^R^ and OHex-H-Mes_C^B^, hexane molecules used for recrystallization were found within the single crystals (Fig. S19 and S20†). The comparison of the two crystal structures of H-Si-Mes (C^R^ and C^B^) revealed that the borepin molecules packed parallelly in C^R^ and orthogonally in C^B^, resulting in different molecular orientations. Only weak intermolecular interactions such as the CH⋯π interactions were observed with no noticeable π–π interactions in either crystal structure, suggesting that excimers were unlikely to have been formed. The C^R^, C^B^, and C^P^ crystals showed slight differences in planarity and bond length in their monomer structures, but a clear correlation between the monomer structure and the RTP properties could not be confirmed.

Taken together, we speculated that the RTP properties may be influenced by the ease of structural relaxation in the T_1_ state in the crystals. To compare the strength of crystal packing, we first compared the densities of the single crystals. However, there was no consistent trend between the density and the RTP properties; whereas the C^R^ crystals of H-Si-Mes and F-H-^F^Mes had lower densities than the corresponding C^B^ or C^P^ crystals, the C^B^ crystal of OHex-H-Mes had a lower density than the C^R^ crystal (Fig. S18–S20†). These single-crystal structures had differing molecular orientations, and because some of them contained hexane molecules, density comparisons might not have provided an accurate assessment of the degree of structural relaxation. For this reason, we analyzed intermolecular interactions by extracting a cluster around one molecule from the single-crystal X-ray structure and performed natural energy decomposition analysis (NEDA) calculations.^25^ The cluster models for NEDA calculations were created by extracting a central molecule and all surrounding molecules that have atoms closer in distance to any atom constituting the central molecule than the sum of their van der Waals radii. The calculation models are shown in Fig. S21–S23.† The analysis results for H-Si-Mes_C^R^ and H-Si-Mes_C^B^ indicated that the total energies of the intermolecular interactions were almost the same (Table 3), suggesting similar crystallization enthalpies within the crystals. However, the nuclear repulsion energy in the C^R^ crystal was approximately half that in the C^B^ crystal. This indicates that in the C^R^ crystal, the nuclei around one molecule are relatively farther apart compared with the C^B^ crystal. In other words, borepin molecules in the C^R^ crystals are more prone to structural relaxation than those in the C^B^ crystals. The relatively lower nuclear repulsion energy in the C^R^ crystal of H-Si-Mes is consistent with the NEDA calculations for F-H-^F^Mes and OHex-H-Mes. Therefore, structural relaxation may occur more easily in the C^R^ crystals than in the corresponding C^B^ or C^P^ crystals; this makes it more likely for RTP properties to appear. In conventional RTP materials, structural relaxation is suppressed by strong intermolecular interactions such as π–π interactions, hydrogen bonding, and halogen bonding, which in turn inhibit thermal deactivation and allow for the expression of RTP properties.^8^ Although a more detailed analysis is required to fully understand the underlying mechanism, the borepin compounds examined in the present study, which exhibit RTP properties that are possibly promoted by structural relaxation, are extremely rare and may provide valuable insights into the molecular design and development of new RTP materials.

**Table 3:** Energy decomposition of cluster models of benzo[*d*]dithieno[*b*,*f*]borepins using NEDA (kcal mol^−1^)

Energy	H-Si-Mes		OHex-H-Mes		F-H-^F^Mes	
	C^R^	C^B^	C^R^	C^B^	C^R^	C^P^
Electrical	−65.8	−75.5	−107.0	−107.9	−62.5	−72.9
Charge transfer	−58.1	−64.3	−92.7	−94.9	−75.8	−81.9
Core	16.4	34.1	66.9	74.0	65.0	80.7
Total interaction	−107.5	−105.7	−132.7	−128.9	−73.2	−74.1

### Preparation of water-dispersible H-Si-Mes with P123 for bioimaging

**Fig. 10:**
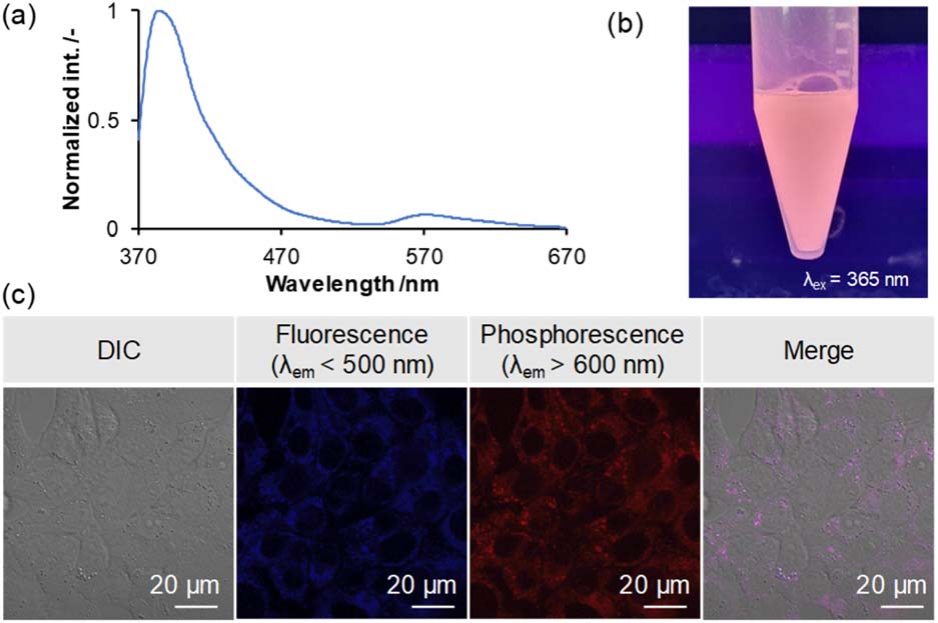
PL spectrum (a) and representative image (b) of H-Si-Mes complex with P123 in aqueous medium. (c) PL signals from the H-Si-Mes complex delivered into the cells. Colon-26 cells were co-incubated with the H-Si-Mes complex for 24 h, and the samples were observed by confocal laser scanning microscopy.

Phosphorescent dyes allow for highly sensitive imaging unaffected by autofluorescence and enable selective imaging in low-oxygen environments, such as cancer cells. Also, there have been many reports of fluorescent bioimaging materials based on triarylboranes in recent years.^12*k*,24,26^ To confirm the utility of the present borepin system with RTP properties as a bioimaging probe, a simple bioimaging experiment was conducted. To use H-Si-Mes as a contrast agent for imaging, it needs to be dispersed in an aqueous medium while maintaining its crystal structure. To this end, we prepared a water-dispersible complex of H-Si-Mes with P123, a triblock polymer comprising polyethylene glycol (PEG) and polypropylene glycol (PPO) (PEG–PPO–PEG), using a top–down method.^27^ The dispersion was analyzed by UV-vis absorption spectra (Fig. S24†) and DLS (Table S9† and Fig. S25†). When the aqueous dispersion was excited at 352 nm, both fluorescence (370–470 nm) and phosphorescence (520–670 nm) bands were observed (Fig. 10a). Moreover, the reddish phosphorescence of the dispersion was confirmed visually (Fig. 10b). These results suggest that H-Si-Mes complexed with P123 maintains its crystal structure even in aqueous medium and that the complex of H-Si-Mes with P123 is applicable as a bioimaging probe. As absorption of H-Si-Mes within P123 micelles and hydrodynamic diameter of the complex did not significantly change even after 3 days from the preparation (Fig. S24 and S25†), suggesting that P123 micelles comprising H-Si-Mes were colloidally stable, and demonstrating the utility of water solubilization of hydrophobic triarylborane photoluminescent dyes using P123. We further examined the encapsulation mechanism using pyrene with P123 (Fig. S26†), and it was found that hydrophobic compounds including H-Si-Mes can be trapped in hydrophobic nanodomain in P123 micelles. Finally, we performed cellular imaging using the complex of H-Si-Me with P123. Murine colon carcinoma (Colon-26) cells were co-incubated with the complex of H-Si-Mes with P123 (H-Si-Me, 6.4 μM) for 24 h, and samples were observed by confocal laser scanning microscopy. The fluorescence (<500 nm) and phosphorescence (>600 nm) could be detected and these two signals were highly overlapped (Fig. 10c). Both luminescent signals were observed within the cells, indicating that the complex was successfully internalized in the cells while maintaining its RTP properties. These results indicate the applicability of the complex as a bioimaging probe *in vitro*.

## Conclusion

In this study, we discovered that benzo[*d*]dithieno[*b*,*f*]borepins exhibit reddish room-temperature phosphorescence. The results of PL spectral analysis and DFT calculations suggested that the low planarity of the tetracyclic structure and the CT transitions between the borepin core and the mesityl group on boron may synergistically contribute to the promotion of ISC and the expression of RTP properties. Although no clear correlation was observed between the single-crystal X-ray structures and the RTP properties, NEDA calculations suggested that structural relaxation in the T_1_ state may enhance RTP properties. Benzo[*d*]dithieno[*b*,*f*]borepins, which do not contain heavy atoms or oxygen atoms in their main framework, notably differ from the molecular structures of RTP materials reported thus far. The insights gained in this study are expected to benefit the development of new RTP materials based on triarylboranes.

## Data availability

All data are available in the manuscript and in the ESl.†

## Author contributions

Yohei Adachi: conceptualization; formal analysis; funding acquisition; investigation; writing – original draft; writing – review & editing. Maho Kurihara: formal analysis; investigation; validation. Kohei Yamada: investigation. Fuka Arai: investigation. Keita Yamana: formal analysis; investigation; validation. Riku Kawasaki: formal analysis; investigation; validation. Joji Ohshita: project administration; supervision; writing – review & editing.

## Conflicts of interest

There are no conflicts to declare.

## Supplementary Material

SC-015-D4SC01184H-s001

SC-015-D4SC01184H-s002

SC-015-D4SC01184H-s003

## References

[cit1] Chou P.-T., Chi Y. (2007). Chem.–Eur. J..

[cit2] Robinson G. W., Frosch R. P. (1963). J. Chem. Phys..

[cit3] Lower S. K., El-Sayed M. A. (1966). Chem. Rev..

[cit4] Ravotto L., Ceroni P. (2017). Coord. Chem. Rev..

[cit5] Kenry, Chen C., Liu B. (2019). Nat. Commun..

[cit6] El-Sayed M. A. (1963). J. Chem. Phys..

[cit7] Xue P., Sun J., Chen P., Wang P., Yao B., Gong P., Zhang Z., Lu R. (2015). Chem. Commun..

[cit8] Nidhankar A. D., Goudappagouda, Wakchaure V. C., Babu S. S. (2021). Chem. Sci..

[cit9] Yuan W. Z., Shen X. Y., Zhao H., Lam J. W. Y., Tang L., Lu P., Wang C., Liu Y., Wang Z., Zheng Q., Sun J. Z., Ma Y., Tang B. Z. (2010). J. Phys. Chem. C.

[cit10] Englman R., Jortner J. (1970). Mol. Phys..

[cit11] Wang X.-F., Guo W.-J., Xiao H., Yang Q.-Z., Chen B., Chen Y.-Z., Tung C.-H., Wu L.-Z. (2020). Adv. Funct. Mater..

[cit12] Ren Y., Jäkle F. (2016). Dalton Trans..

[cit13] Wu Z., Nitsch J., Schuster J., Friedrich A., Edkins K., Loebnitz M., Dinkelbach F., Stepanenko V., Würthner F., Marian C. M., Ji L., Marder T. B. (2020). Angew. Chem., Int. Ed..

[cit14] Adachi Y., Ohshita J. (2018). Organometallics.

[cit15] Adachi Y., Arai F., Jäkle F. (2020). Chem. Commun..

[cit16] Yin X., Chen J., Lalancette R. A., Marder T. B., Jäkle F. (2014). Angew. Chem., Int. Ed..

[cit17] Jia W.-L., Song D., Wang S. (2003). J. Org. Chem..

[cit18] Dong Y., Sukhanov A. A., Zhao J., Elmali A., Li X., Dick B., Karatay A., Voronkova V. K. (2019). J. Phys. Chem. C.

[cit19] Narsaria A. K., Rauch F., Krebs J., Endres P., Friedrich A., Krummenacher I., Braunschweig H., Finze M., Nitsch J., Bickelhaupt F. M., Marder T. B. (2020). Adv. Funct. Mater..

[cit20] Luciano H. M., Farias G., Salla C. M., Franca L. G., Kuila S., Monkman A. P., Durola F., Bechtold I. H., Bock H., Gallardo H. (2023). Chem.–Eur. J..

[cit21] Nagarajan K., Mallia A. R., Muraleedharan K., Hariharan M. (2017). Chem. Sci..

[cit22] Levine D. R., Siegler M. A., Tovar J. D. (2014). J. Am. Chem. Soc..

[cit23] Mercier L. G., Furukawa S., Piers W. E., Wakamiya A., Yamaguchi S., Parvez M., Harrington R. W., Clegg W. (2011). Organometallics.

[cit24] Ferger M., Roger C., Köster E., Rauch F., Lorenzen S., Krummenacher I., Friedrich A., Košćak M., Nestić D., Braunschweig H., Lambert C., Piantanida I., Marder T. B. (2022). Chem.–Eur. J..

[cit25] Glendening E. D., Streitwieser A. (1994). J. Chem. Phys..

[cit26] Griesbeck S., Ferger M., Czernetzi C., Wang C., Bertermann R., Friedrich A., Haehnel M., Sieh D., Taki M., Yamaguchi S., Marder T. B. (2019). Chem.–Eur. J..

[cit27] Zhen X., Tao Y., An Z., Chen P., Xu C., Chen R., Huang W., Pu K. (2017). Adv. Mater..

