# Supplementary material for: Insights into mechanistic interpretation of crystalline-state reddish phosphorescence of non-planar π-conjugated organoboron compounds
Source: Chem Sci. 2024 Apr 25;15(21):8127–36. doi: 10.1039/d4sc01184h (PMC11134383; doi:10.1039/d4sc01184h)
Supplement: SC-015-D4SC01184H-s001 [file SC-015-D4SC01184H-s001.pdf]

Electronic Supplementary Material (ESI) for Chemical Science.
This journal is © The Royal Society of Chemistry 2024

Supporting Information

Insights into mechanistic interpretation of crystalline-state reddish phosphorescence of non-planar π-conjugated organoboron compounds

Yohei Adachi,*[a] Maho Kurihara,[a] Kohei Yamada,[a] Fuka Arai,[a] Yuto Hattori,[a] Keita Yamana,[b] Riku Kawasaki[b] and Joji Ohshita*[a,c]

[a] *Smart Innovation Program, Graduate School of Advanced Science and Engineering, Hiroshima University, Higashi-Hiroshima 739-8527, Japan. E-mail: yadachi@hiroshima-u.ac.jp, jo@hiroshima-u.ac.jp*

[b] *Applied Chemistry Program, Graduate School of Advanced Science and Engineering, Hiroshima University, Higashi-Hiroshima 739-8527, Japan.*

[c] *Division of Materials Model-Based Research, Digital Monozukuri (Manufacturing) Education and Research Center, Hiroshima University, Higashi-Hiroshima, Hiroshima 739-8527, Japan.*

Experimental

Materials

All reactions were carried out under dry argon. For the reaction solvents, diethyl ether and toluene were purchased from Kanto Chemical Co., Ltd., distilled from calcium hydride, and stored over activated molecular sieves under argon until use. All other chemicals were purchased from FUJIFILM Wako Pure Chemical Industries, Ltd. and TCI Co., Ltd. Starting materials **6**,[S1] **1-H**,[S2] and **1-F**[S2] were prepared according to the literature procedure. Colon26 cells were kindly supplied by Prof. Nagasaki (Osaka Metropolitan University). The cells were maintained in Dulbecco's Modified Eagle Medium (Thermo Fischer Scientifc, Massachusetts, USA) containing 10% fatal bovine serum (Thermo Fischer Scientific) and antibiotics (Thermo Fischer Scientific).

Analytical Methods

NMR spectra were recorded on Varian System 500 and 400MR spectrometers. Abbreviations Th, Bz, Mes, Tipp, and ^{F}Mes used for the following NMR assignments stand for fused thiophene ring, fused benzene ring, mesity group, tripyl group, and 2,4,6-tris(trifluoromethylphenyl) group, respectively. High-resolution mass spectra were obtained by the direct infusion method on a Thermo Fisher Scientific LTQ Orbitrap XL spectrometer at N-BARD, Hiroshima University. UV–vis absorption spectra were measured with a Shimadzu UV-3600 Plus spectrometer. Photoluminescence (PL) spectra, phosphorescence lifetimes, and absolute PL quantum yields were measured with a HORIBA FluoroMax-4 spectrophotometer with an integrating sphere. Fluorescence lifetimes were on a HORIBA DeltaFlex modular fluorescence lifetime system, using a Nano LED pulsed diode excitation source. Variable temperature emission spectra and lifetimes were recorded on a HORIBA Fluorolog-3 spectrofluorometer and corrected for the response of the detector system.

DFT calculations

The S_0 geometries were obtained by DFT calculations using a Gaussian 16 program at the B3LYP/6-31G(d) level of theory. The S_1 and T_1 geometries were obtained by TD-DFT calculations at the same level. All the

optimized geometries were verified by vibrational frequency analysis at the same level of theory and found as true minima, as negative vibrational frequencies were not present in all cases. All the TD-DFT calculations were performed for the 10 lowest singlet and triplet states on the S_0 and T_1 geometries. Spin-orbit coupling matrix elements (SOCMEs) were calculated from TD-DFT results at the B3LYP/6-31G(d) level for the ten lowest singlet and triplet transitions by using the Orca 5.0.3 package software. NEDA calculations were performed on Gaussian 16 with NBO version 7.0. Due to limitations on the number of shells in NBO calculations, it was impossible to perform NEDA calculations for all molecules in the cluster models shown in Figures S15-S17. Therefore, NEDA calculations were conducted for models divided into groups of 2 to 3 molecules, consisting of the central molecule and its surrounding molecules. Finally, the sum of the calculated energies was obtained from the results of each calculation. Dividing the models may not be appropriate when there are strong intermolecular interactions between the divided molecules. However, in the cases studied in this work, since there are no strong intermolecular interactions between the molecules, the division did not significantly impact the computational results. In fact, even when the model was divided with different combinations of molecules, the computational results did not change significantly.

XRD measurements

Single crystal X-ray diffraction data was collected at 100 K on a Bruker AXS SMART APEX II ULTRA diffractometer or on a Rigaku XtaLAB Synergy R, DW diffractometer at Natural Science Center for Basic Research and Development (N-BARD), Hiroshima University, using MoKα radiation monochromated with a multilayered confocal mirror. The structure was solved by Intrinsic Phasing on the SHELXT-2014/5 program or by the CrysAlis Pro and expanded using Fourier techniques. Non-hydrogen atoms were refined anisotropically, whereas hydrogen atoms were included but not refined (SHELXL-2017/1). All other calculations were performed using the APEXII crystallographic software package of Bruker AXS or using the OLEX2 program. Graphical crystal structures were generated using Mercury 4.3.1 (Cambridge Crystallographic Data Centre).

*Preparation of water-dispersible **H-Si-Mes** and pyrene*

The crystal of **H-Si-Mes** or pyrene were co-incubated with P123 in MilliQ (**H-Si-Mes** or pyrene 0.5 mg; P123, 4.5 mg) and the resulting solution was sonicated (60 W, 5 min) with probe type sonicator on ice. After removal of precipitation by centrifugation (4 °C, 10000 g, 15 min), the dispersibility was confirmed by measuring UV-Vis absorption spectrum. We further addressed size of the complex of **H-Si-Mes** with P123 by dynamic light scattering (DLS) measurement (Zeta Sizer Nano, Malvern, UK). In addition, their electrochemically negative character was also revealed by ζ-potential measurement. Morphological observation was carried out by transmission electron microscope (TEM, JEM-1400, JEOL Ltd. Co., Japan). The samples were casted on the grid and stained with 4 % phosphotungstic acid. The stained samples were observed by TEM (acceleration voltage, 100 keV).

Bioimaging

Murine colon carcinoma cells were exposed to the complex of **H-Si-Mes** with P123 for 24 h. After washing with PBS, the medium were exchanged with fresh medium. The samples were observed by confocal laser scanning microscopy (LSM700, Carl Zeiss, Germany).

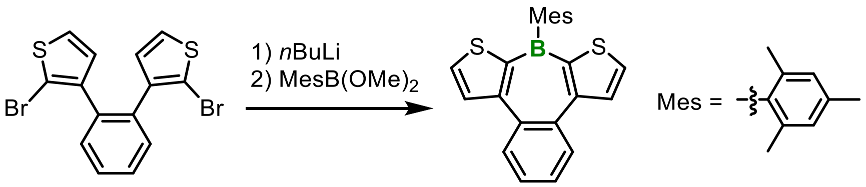

6 **H-H-Mes**

*Synthesis of **H-H-Mes***

To a solution of 1.00 g (2.50 mmol) of **6** in 9.5 mL of toluene and 2.5 mL of diethyl ether was added 3.30 mL (5.13 mmol) of 1.55 mol/L *n*BuLi in hexane at –78 °C over a period of 5 min and the mixture was stirred at this temperature for 20 min. To this was added 528 mg (2.75 mmol) of $MesB(OMe)_2$ at this temperature and the mixture was stirred at room temperature overnight. The resulting mixture was hydrolyzed with saturated NH_4Cl aqueous solution, and 30 mL of toluene was added. The organic layer was separated, and the aqueous layer was extracted with 30 mL of toluene. The combined organic layers were washed twice with water and then once with brine. After drying over anhydrous magnesium sulfate, the solvent was evaporated. The crude

product was purified by silica gel chromatography using a mixed solvent of *n*-hexane/dichloromethane = 4/1 as the eluent to give 504 mg (1.36 mmol, 54% yield) of **H-H-Mes** as a white solid. Crystals obtained by recrystallization from a hexane solution of the above product were used for optical and X-ray analysis. ^{1}H NMR (500 MHz, $CDCl_3$) δ: 8.38 (dd, *J* = 6.2, 3.5 Hz, 2H, Bz), 8.13 (d, *J* = 5.1 Hz, 2H, Th), 7.94 (d, *J* = 5.0 Hz, 2H, Th), 7.59 (dd, *J* = 6.2, 3.4 Hz, 2H, Bz), 6.93 (d, *J* = 0.4 Hz, 2H, Mes), 2.39 (s, 3H, Mes), 2.10 (s, 6H, Mes). ^{13}C{^{1}H} NMR (126 MHz, $CDCl_3$) δ: 150.8, 144.4 (br, B–C), 140.9 (br, B–C), 138.8, 137.5, 135.2, 133.1, 131.5, 131.2, 127.5, 127.1, 22.8, 21.4. ^{11}B NMR (160 MHz, $CDCl_3$) δ: 50.4. HRMS (APCI) Calcd. for $C_{23}H_{19}BClS_2$: $[M+Cl]^-$: 405.07152, Found: 405.07166. m.p. 177.3-179.0 °C.

Synthesis of **F-H-Mes** has been already reported in our previous work.[S1]

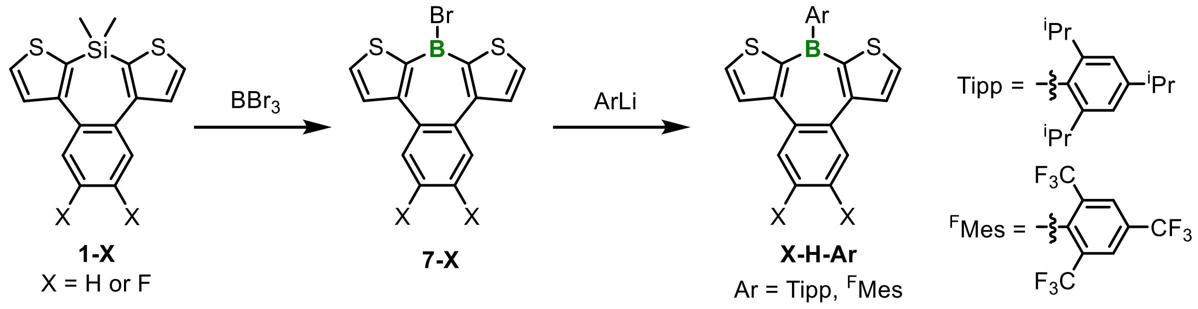

Synthesis of **7-X** from **1-X** has been already reported in our previous work.[S2]

*Synthesis of **H-H-Tipp***

To a solution of 1-bromo-2,4,6-triisopropylbenzene (0.671 g, 2.37 mmol) in 15 mL of diethyl ether was slowly added 3.05 mL (4.64 mmol) of 1.52 mol/L *t*BuLi in pentane at –78 °C. The mixture was stirred for 1 hour at the temperature, then the solvent was removed in vacuum at 0 °C to give the lithium salt. The solid was redissolved in 7 mL of toluene at 0 °C, then cooled to –78 °C. A solution of **6-H** (0.730 g, 2.21 mmol) in 8 mL of toluene was slowly added to the above solution, then the mixture was stirred overnight at room temperature. The resulting mixture was hydrolyzed with saturated NH_4Cl aqueous solution, and the organic layer was washed twice with brine. After drying over anhydrous magnesium sulfate, the solvent was evaporated. The crude product was purified by silica gel chromatography using a mixed solvent of *n*-hexane/dichloromethane = 4/1 as the eluent to give 614 mg (1.35 mmol, 61% yield) of the title compound as a white solid. Crystals obtained by recrystallization from a hexane solution of the above product were used

for optical and X-ray analysis. ^{1}H NMR (500 MHz, $CDCl_3$) δ: 8.41 (dd, J = 6.2, 3.5 Hz, 2H, Bz), 8.16 (d, J = 5.1 Hz, 2H, Th), 7.92 (d, J = 5.1 Hz, 2H, Th), 7.59 (dd, J = 6.2, 3.4 Hz, 2H, Bz), 7.05 (s, 2H, Tipp), 2.98 (hept, J = 7.0 Hz, 1H, Tipp), 2.42 (hept, J = 6.7 Hz, 2H, Tipp), 1.34 (d, J = 6.9 Hz, 6H, Tipp), 1.10 (d, J = 6.7 Hz, 12H, Tipp). ^{13}C{^{1}H} NMR (126 MHz, $CDCl_3$) δ: 150.2, 150.1, 148.9, 145.6 (br, B–C), 138.6 (br, B–C), 134.9, 133.1, 131.6, 130.9, 127.4, 120.2, 35.4, 34.2, 24.8, 24.1. ^{11}B NMR (160 MHz, $CDCl_3$) δ 50.4. HRMS (APCI) Calcd. for $C_{29}H_{32}BS_2$: $[M+H]^+$: 455.20330, Found: 455.20309. m.p. 207.2-207.6 °C.

Synthesis of ***F-H-Tipp***

F-H-Tipp was prepared from 0.385 g (1.36 mmol) of 1-bromo-2,4,6-triisopropylbenzene, 1.63 mL (2.48 mmol) of 1.52 mol/L *t*BuLi in pentane, 0.415 g (1.13 mmol) of **6-F**, 10 mL of diethyl ether as a white solid (308 mg, 0.628 mmol, 56% yield) in a manner similar to that above. ^{1}H NMR (500 MHz, $CDCl_3$) δ: 8.17 (t, $J_{C\text{-}F}$ = 10.8 Hz, 2H, Bz), 8.01 (d, J = 5.1 Hz, 2H, Th), 7.94 (d, J = 5.1 Hz, 2H, Th), 7.05 (s, 2H, Tipp), 2.99 (hept, J = 6.9 Hz, 1H, Tipp), 2.37 (hept, J = 6.7 Hz, 2H, Tipp), 1.34 (d, J = 6.9 Hz, 6H, Tipp), 1.10 (d, J = 6.7 Hz, 12H, Tipp). ^{13}C{^{1}H} NMR (126 MHz, $CDCl_3$) δ: 150.1, 149.1, 148.8 (dd, $J_{C\text{-}F}$ = 251.8, 15.4 Hz), 148.2, 135.5, 130.8, 130.5 (t, $J_{C\text{-}F}$ = 4.3 Hz), 120.3, 119.6 (dd, $J_{C\text{-}F}$ = 12.6, 6.8 Hz), 35.5, 34.2, 24.7, 24.1. Two signals for B−C were not detected, probably due to its low intensity as a result of quadrupolar broadening. ^{11}B NMR (160 MHz, $CDCl_3$) δ: 50.3. HRMS (APCI) Calcd. for $C_{29}H_{29}BClF_2S_2$: $[M+Cl]^-$: 525.14658, Found: 525.14752. m.p. 202.0-202.3 °C.

Synthesis of ***H-H-^{F}Mes***

To a solution of 1,3,5-tris(trifluoromethyl)benzene (0.677 g, 2.40 mmol) in 15 mL of diethyl ether was slowly added 1.40 mL (2.23 mmol) of 1.59 mol/L *n*BuLi in hexane at –78 °C. The mixture was stirred for 3 hours at room temperature, then the solvent was removed in vacuum to give the lithium salt. The solid was redissolved in 7 mL of toluene at 0 °C, then cooled to –78 °C. A solution of **6-H** (0.725 g, 2.19 mmol) in 8 mL of toluene was slowly added to the above solution, then the mixture was stirred overnight at room temperature. The resulting mixture was hydrolyzed with saturated NH_4Cl aqueous solution, and the organic layer was washed twice with brine. After drying over anhydrous magnesium sulfate, the solvent was

evaporated. The crude product was purified by silica gel chromatography using *n*-hexane as the eluent to give 466 mg (0.876 mmol, 40% yield) of the title compound as a white solid. Crystals obtained by recrystallization from a hexane solution of the above product were used for optical and X-ray analysis. ^{1}H NMR (500 MHz, $CDCl_3$) δ: 8.38 (dd, J = 6.2, 3.5 Hz, 2H, Bz), 8.19 (s, 2H, ^{F}Mes), 8.14 (d, J = 5.1 Hz, 2H, Th), 7.96 (d, J = 5.0 Hz, 2H, Th), 7.61 (dd, J = 6.2, 3.4 Hz, 2H, Bz). ^{13}C{^{1}H} NMR (126 MHz, $CDCl_3$) δ: 152.0, 143.8 (br, B–C), 141.3 (br, B–C), 135.0, 134.9 (q, J = 31.9 Hz, *o*-<u>C</u>CF_3), 132.8, 132.0 (q, J = 34.5 Hz, *p*-<u>C</u>CF_3), 131.7, 131.2, 127.9, 126.4 (br), 123.6 (q, J = 275.6 Hz, *o*-CF_3), 122.9 (q, J = 272.8 Hz, *p*-CF_3). ^{11}B NMR (160 MHz, $CDCl_3$) δ: 47.2. HRMS (APCI) Calcd. for $C_{23}H_{11}BF_9S_2$: M^+: 533.02460, Found: 533.02411. m.p. 157.2-157.9 °C.

Synthesis of ***F-H-^{F}Mes***

F-H-^{F}Mes was prepared from 0.705 g (2.50 mmol) of 1,3,5-tris(trifluoromethyl)benzene, 1.46 mL (2.32 mmol) of 1.59 mol/L *n*BuLi in hexane, 0.852 g (2.32 mmol) of **6-F**, 15 mL of diethyl ether as a white solid (451 mg, 0.794 mmol, 34% yield) in a manner similar to that above. ^{1}H NMR (500 MHz, $CDCl_3$) δ: 8.19 (s, 2H, ^{F}Mes), 8.14 (t, $J_{C\text{-}F}$ = 10.6 Hz, 2H, Bz), 8.00 (d, J = 5.1 Hz, 2H, Th), 7.98 (d, J = 5.1 Hz, 2H, Th). ^{13}C{^{1}H} NMR (126 MHz, $CDCl_3$) δ: 150.0, 149.0 (dd, $J_{C\text{-}F}$ = 253.0, 15.1 Hz), 143.2 (br, B–C), 141.5 (br, B–C), 135.6, 134.9 (q, $J_{C\text{-}F}$ = 32.0 Hz, *o*-<u>C</u>CF_3), 132.2 (q, $J_{C\text{-}F}$ = 34.5 Hz, *p*-<u>C</u>CF_3), 131.0, 130.2 (t, $J_{C\text{-}F}$ = 4.4 Hz), 126.4 (br), 123.5 (q, $J_{C\text{-}F}$ = 275.6 Hz, *o*-CF_3), 122.8 (q, $J_{C\text{-}F}$ = 272.8 Hz, *p*-CF_3) , 119.8 (dd, $J_{C\text{-}F}$ = 12.9, 7.1 Hz). ^{11}B NMR (160 MHz, $CDCl_3$) δ: 47.8. HRMS (APCI) Calcd. for $C_{23}H_8BClF_{11}S_2$: M^+: 602.96788, Found: 602.96832. m.p. 171.1-171.6 °C.

__Figures and Tables__

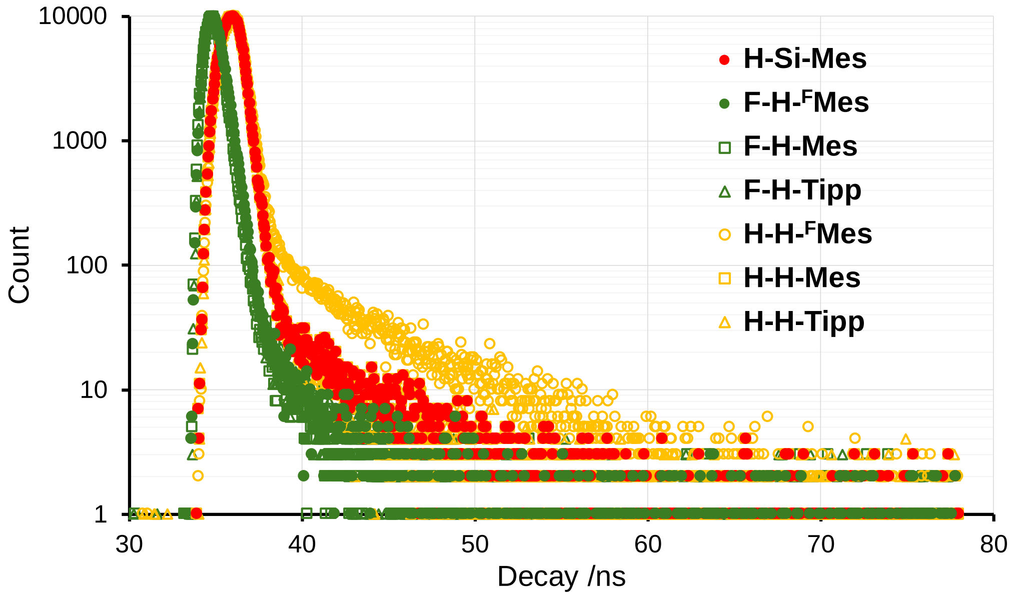

Figure S1 Fluorescence decays of benzo[*d*]dithieno[*b*,*f*]borepins in THF at room temperature.

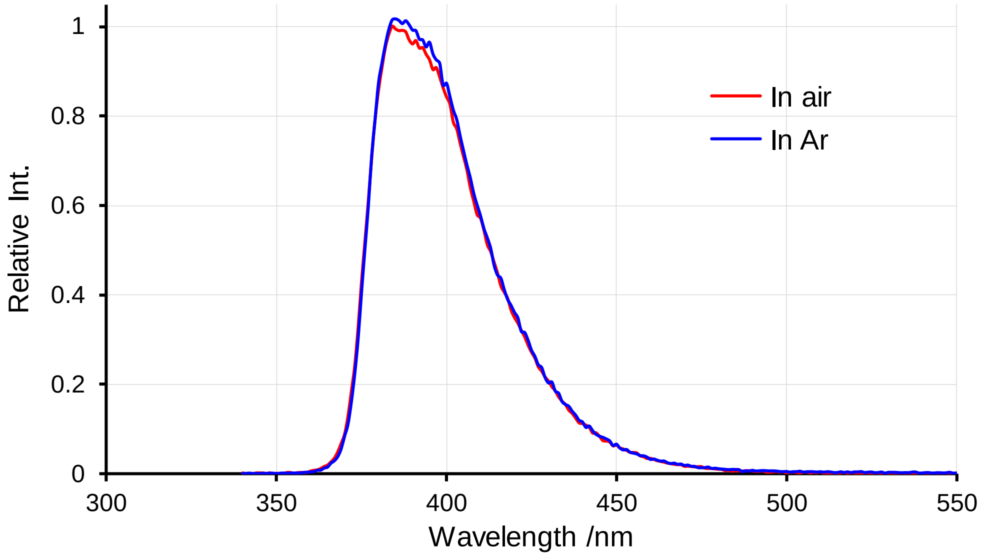

Figure S2 Fluorescence spectra of **H-Si-Mes** before and after argon bubbling in THF at room temperature.

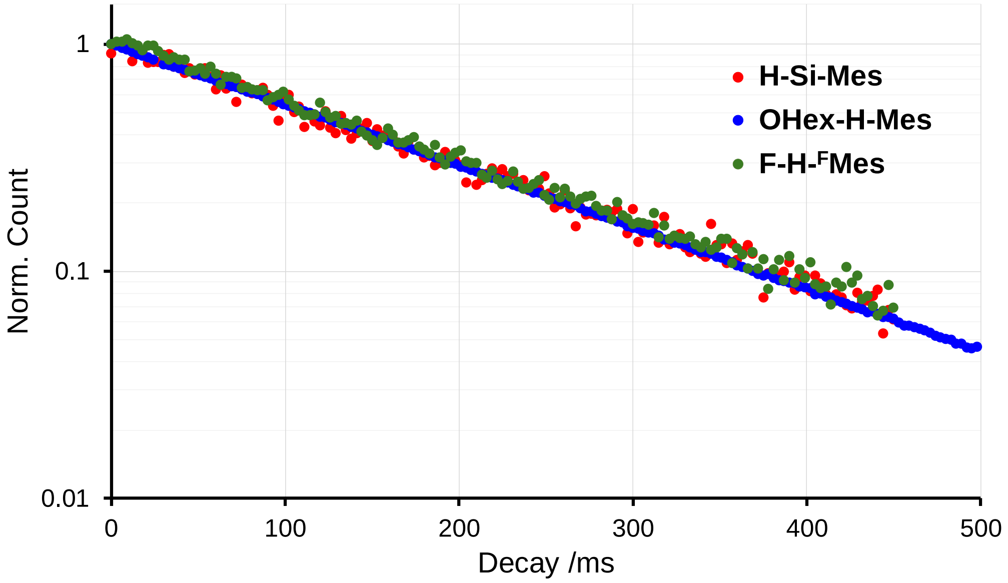

Figure S3 Phosphorescence decays of benzo[*d*]dithieno[*b*,*f*]borepins in 2-THF glass matrix at 77 K.

Compound	Crystal	Amorphous
**F-H-Mes**	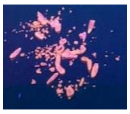	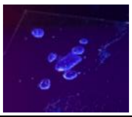
**F-H-Tipp**	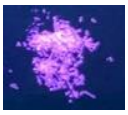	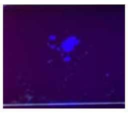
**H-H-FMes**	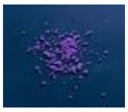	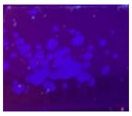
**H-H-Mes**	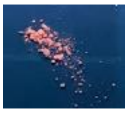	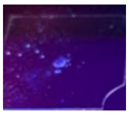
**H-H-Tipp**	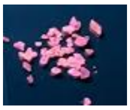	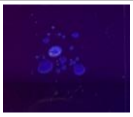

Figure S4 Photographs of recrystallized crystals of benzo[*d*]dithieno[*b*,*f*]borepins taken under room light or UV (365 nm).

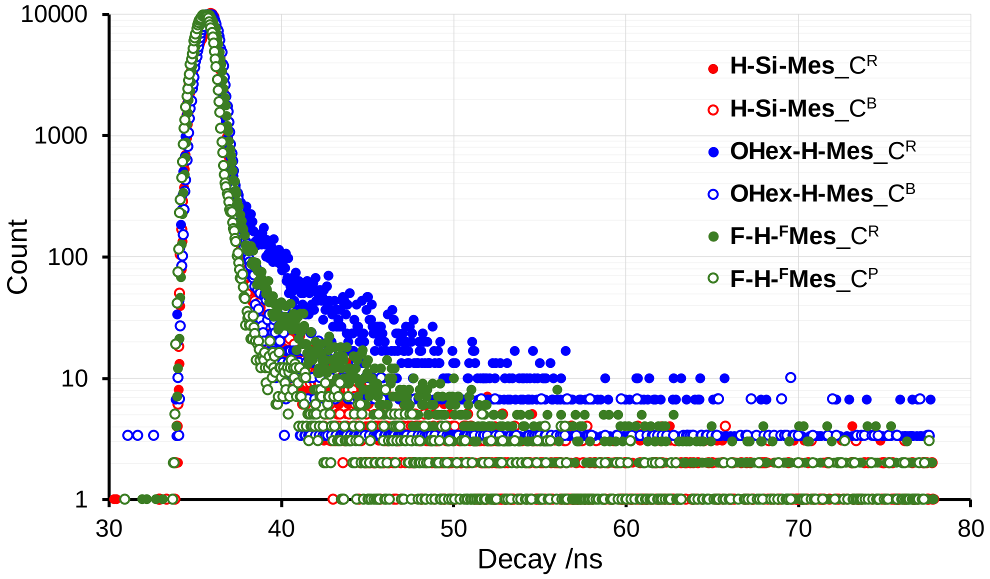

Figure S5 Fluorescence decays of benzo[*d*]dithieno[*b*,*f*]borepins in the solid state at room temperature.

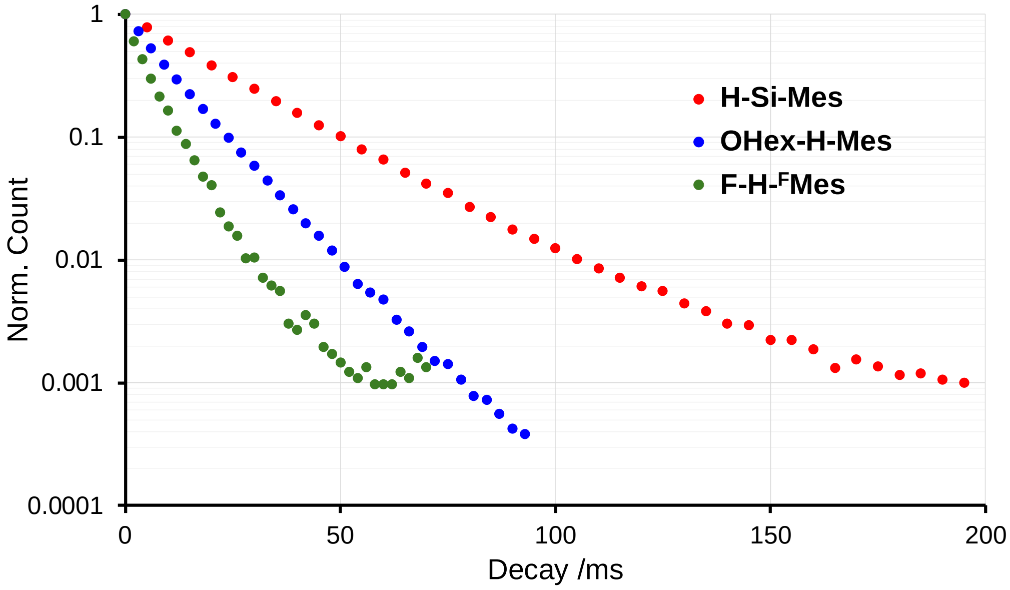

Figure S6 Phosphorescence decays of benzo[*d*]dithieno[*b*,*f*]borepins in the solid state at room temperature.

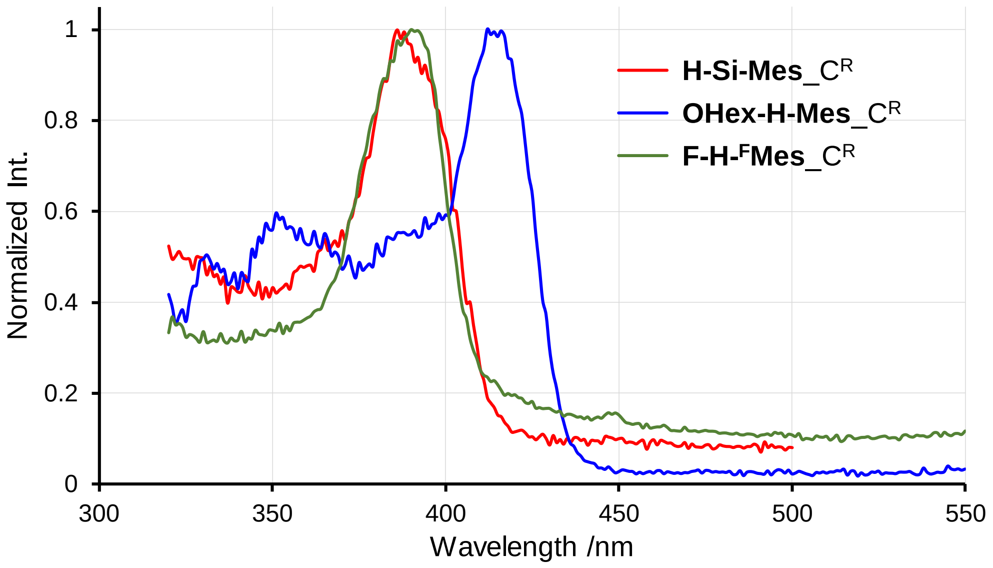

Figure S7 Excitation spectra of the C^R crystals of the borepins in air at room temperature monitored at 600 nm.

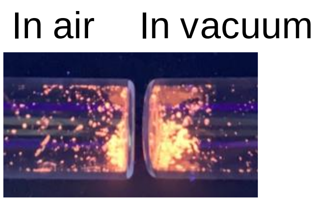

Figure S8 Photographs of **H-Si-Mes**_C^R taken under UV (365 nm) in air and vacuum.

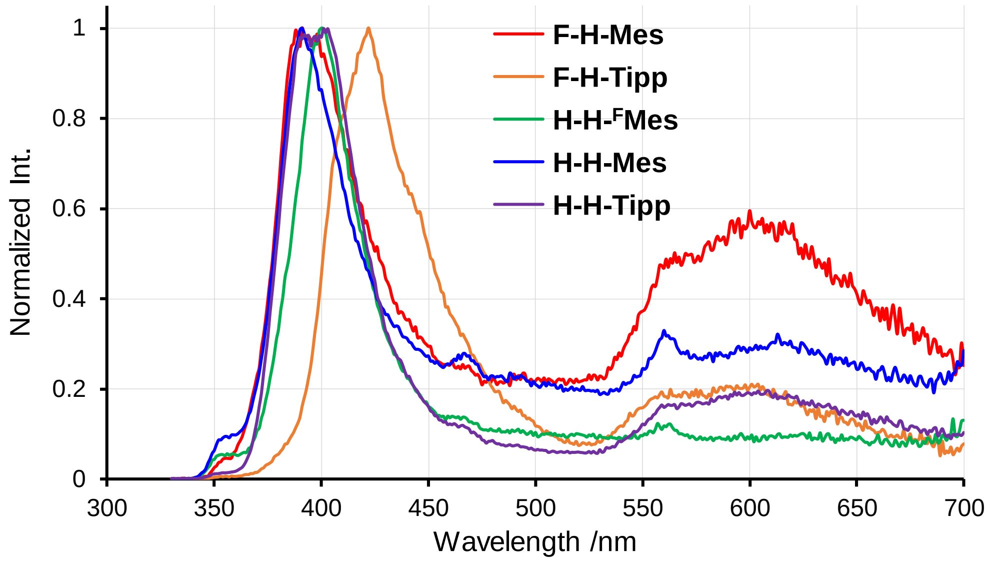

Figure S9 PL spectra of crystals of **X-H-Ar** in air at room temperature.

Table S1 Optical data of **X-H-Ar** in the solid state.

Compound	λ^{FL}[a] /nm	λ^{Phos}[b] /nm	Φ_{PL}[c]	I^{Phos}/I^{Fl}[d]
**F-H-Mes**	391	600	<0.02	0.60
**F-H-Tipp**	422	604	<0.02	0.21
**H-H-^{F}Mes**	400	557	<0.02	0.12
**H-H-Mes**	391	560	<0.02	0.33
**H-H-Tipp**	401	608	<0.02	0.20

[a] Fluorescence maximum. [b] Phosphorescence maximum. [c] Absolute PL quantum yield at room temperature. [d] Intensity ratio of fluorescent and phosphorescent bands.

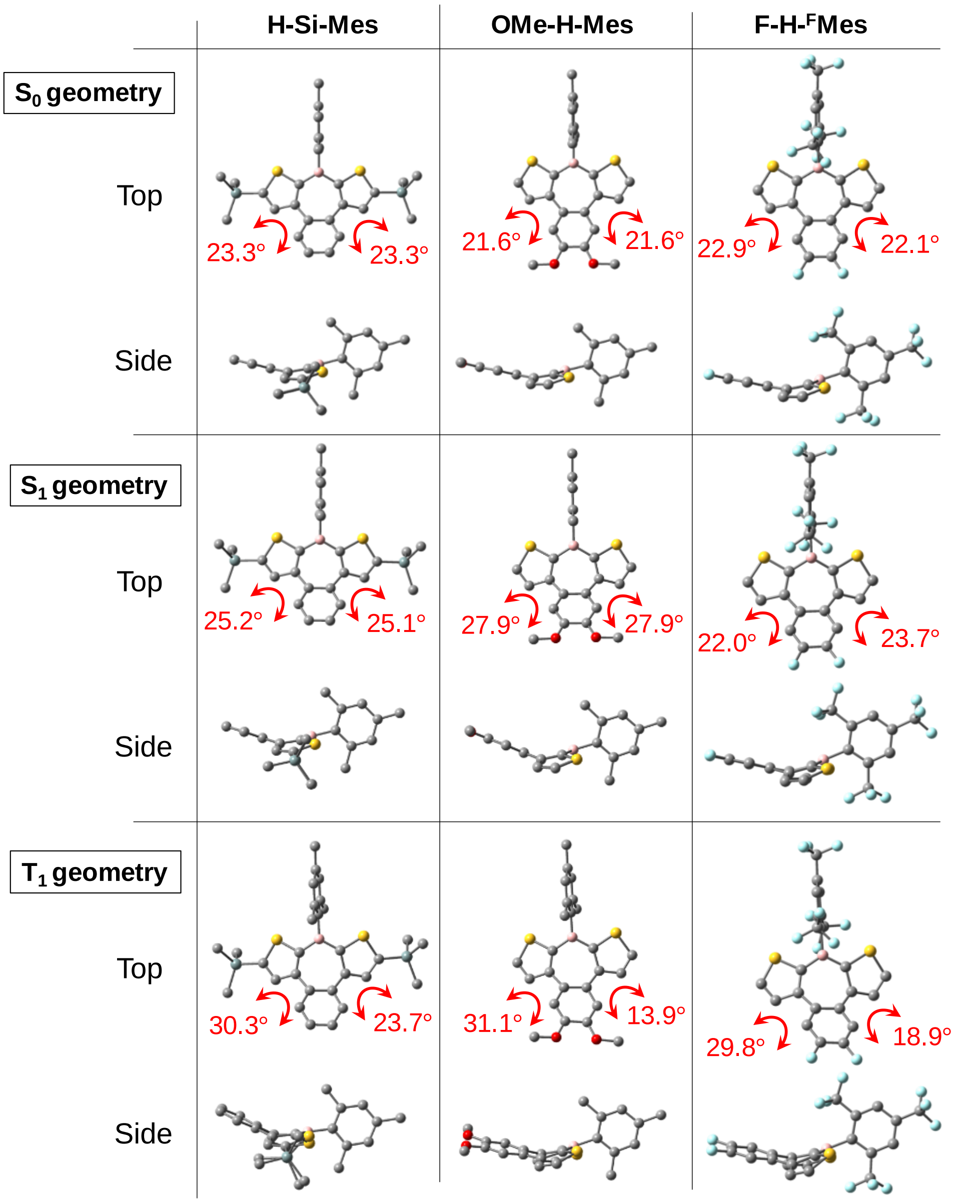

Figure S10 Optimized structures of benzo[*d*]dithieno[*b*,*f*]borepins with dihedral angles of C=C-C=C in S_0 , S_1, and T_1 geometries at the B3LYP/6-31G(d) level.

Table S2 TD-DFT results of benzo[*d*]dithieno[*b*,*f*]borepins in T_1 geometry at the B3LYP/6-31G(d) level.

	Excited State	Composition	CI	Excitation energy /eV	Wavelength /nm	Oscillator strength
**H-Si-Mes**	S0→S1	H→L	0.700	3.27	379	0.00
	S0→S2	H-1→L	0.677	3.50	354	0.09
	S0→T1	H-3→L	0.369	2.66	467	0.00
		H-2→L	0.486			
	S0→T2	H-1→L	0.652	2.82	439	0.00
	S0→T3	H→L	0.566	3.21	387	0.00
	S0→T4	H-4→L	-0.375	3.30	376	0.00
		H-1→L+1	-0.329			
		H→L	0.383			
	S0→T5	H-5→L	0.476	3.38	367	0.00
		H-2→L+1	-0.271			
		H-1→L	-0.231			
**OMe-H-Mes**	S0→S1	H→L	0.688	3.27	379	0.18
	S0→S2	H-1→L	0.701	3.43	362	0.00
	S0→T1	H→L	0.676	2.70	460	0.00
	S0→T2	H-2→L	0.512	2.73	454	0.00
		H→L+1	-0.360			
	S0→T3	H-4→L	-0.255	3.19	388	0.00
		H-2→L	0.266			
		H→L+1	0.535			
	S0→T4	H-1→L	0.655	3.40	365	0.00
	S0→T5	H-6→L	0.381	3.46	359	0.00
		H-2→L+1	0.419			
**F-H-^{F}Mes**	S0→S1	H→L	0.676	3.55	349	0.12
	S0→S2	H-1→L	0.672	3.77	329	0.08
	S0→T1	H-1→L	0.604	2.74	453	0.00
		H→L+3	-0.282			
	S0→T2	H→L	0.649	2.88	431	0.00
	S0→T3	H-2→L	0.385	3.31	375	0.00
		H→L+3	0.465			
	S0→T4	H-3→L	0.426	3.47	357	0.00
		H-1→L+3	-0.403			
	S0→T5	H-6→L+2	0.353	3.59	345	0.00
		H-4→L+1	0.482			

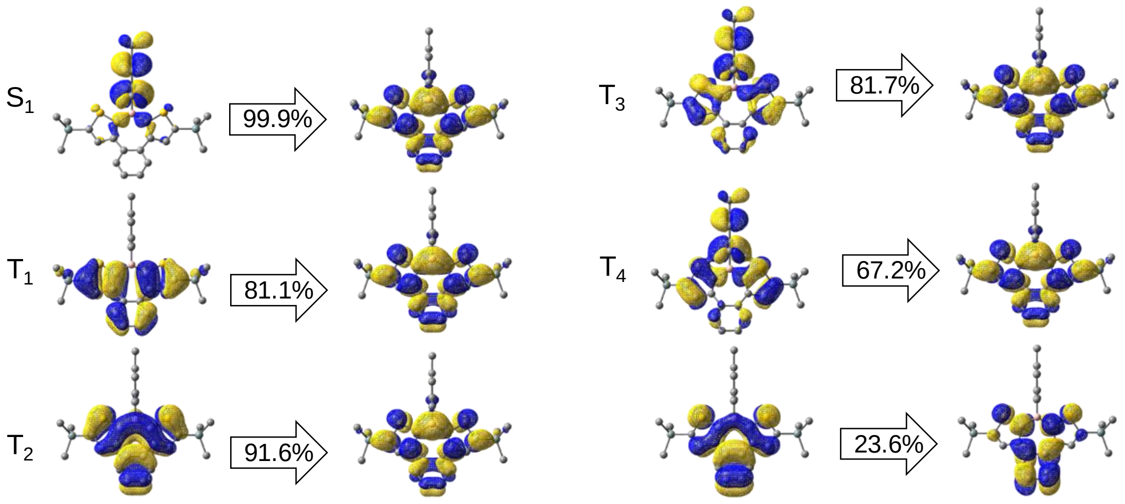

Figure S11 NTOs of **H-Si-Mes** at the B3LYP/6-31G(d) level of theory.

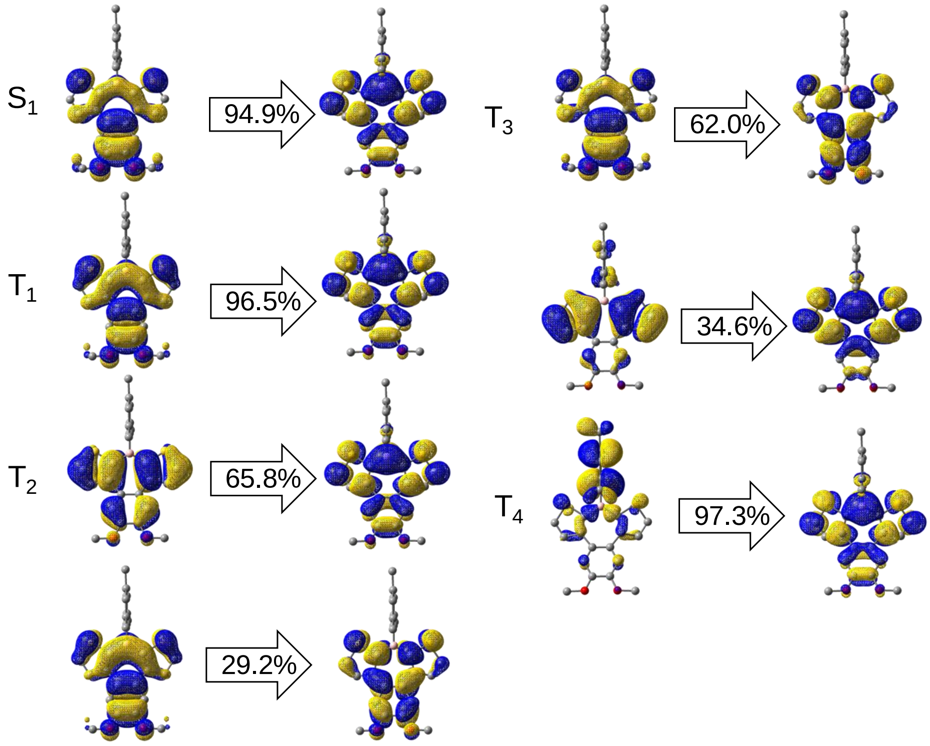

Figure S12 NTOs of **H-Si-Mes** at the B3LYP/6-31G(d) level of theory.

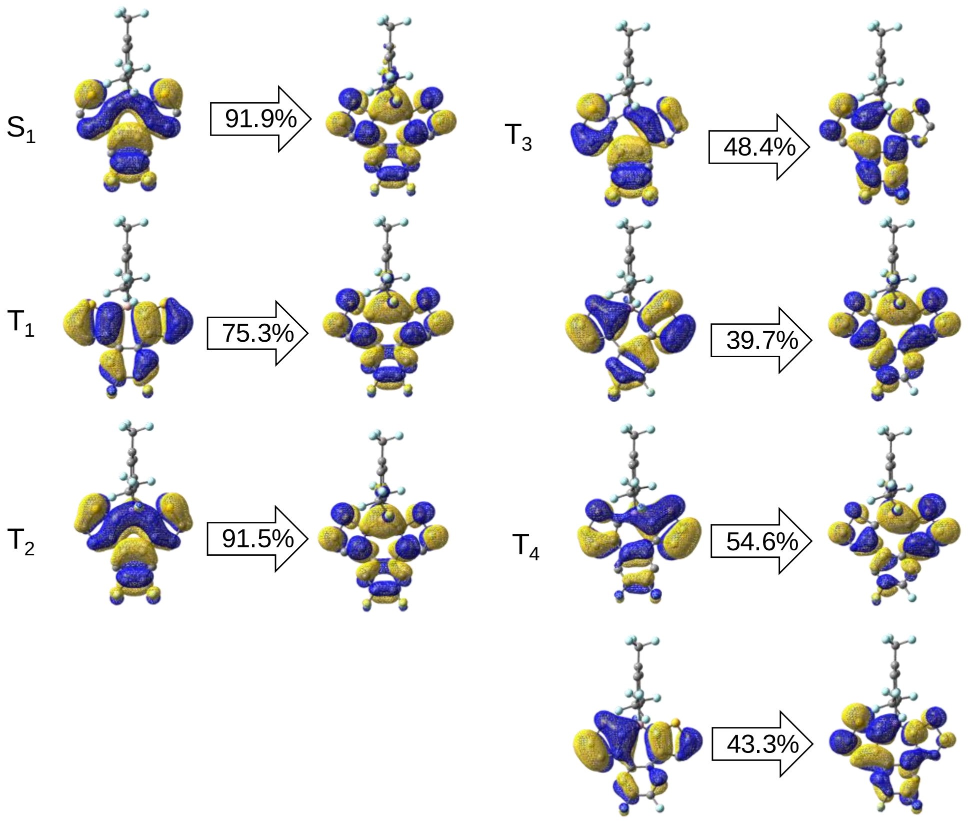

Figure S13 NTOs of **H-Si-Mes** at the B3LYP/6-31G(d) level of theory.

Table S3 SOCMEs (cm^{-1}) and ΔE_{ST} (eV) in S_0 geometry for benzo[*d*]dithieno[*b*,*f*]borepins and model compounds at the B3LYP/6-31G(d) level. The chemical structures of model compounds **2-5** are shown in Figure S8.

State		**H-Si-Mes**	**OMe-H-Mes**	**F-H-^{F}Mes**	**H-H-Mes**	**1-H**	**2**	**3**	**4**	**5**
T_n	S_n									
1	0	3.30	5.54	3.11	3.29	1.25	0.39	2.17	0.24	0.28
1	1	1.16	0.10	1.94	1.06	5.67	0.00	1.47	0.03	0.92
2	1	5.55	2.44	0.30	5.33	0.14	0.00	0.17	0.00	5.42
3	1	4.52	2.71	2.44	3.88	0.73	0.16	3.06	0.04	3.72
4	1	4.89	5.88	0.35	3.51	1.96	0.06	0.05	0.01	1.98
5	1	4.03	0.11	0.45	4.24	0.31	0.08	3.93	0.01	0.00
$\sum_{n=1}^{5} \lvert\langle S_1 \lvert \hat{H}_{SO} \rvert T_n \rangle\rvert$		20.15	11.24	5.48	18.02	8.81	0.30	8.68	0.09	12.04
ΔE_{ST}		0.62	0.57	0.81	0.63	1.29	1.33	0.93	1.11	0.92

Table S4 SOCMEs (cm^{-1}) in S_1 geometry for benzo[*d*]dithieno[*b*,*f*]borepins at the B3LYP/6-31G(d) level.

State T_n	State S_n	H-Si-Mes	OMe-H-Mes	F-H-^{F}Mes
1	0	4.61	8.37	4.71
1	1	0.61	0.25	2.06
2	1	3.87	2.63	0.63
3	1	0.77	4.85	3.73
4	1	6.67	5.51	0.16
5	1	1.24	0.31	3.08
$\sum_{n=1}^{5}\lvert\langle S_1\lvert\hat{H}_{SO}\rvert T_n\rangle\rvert$		13.16	13.55	9.66

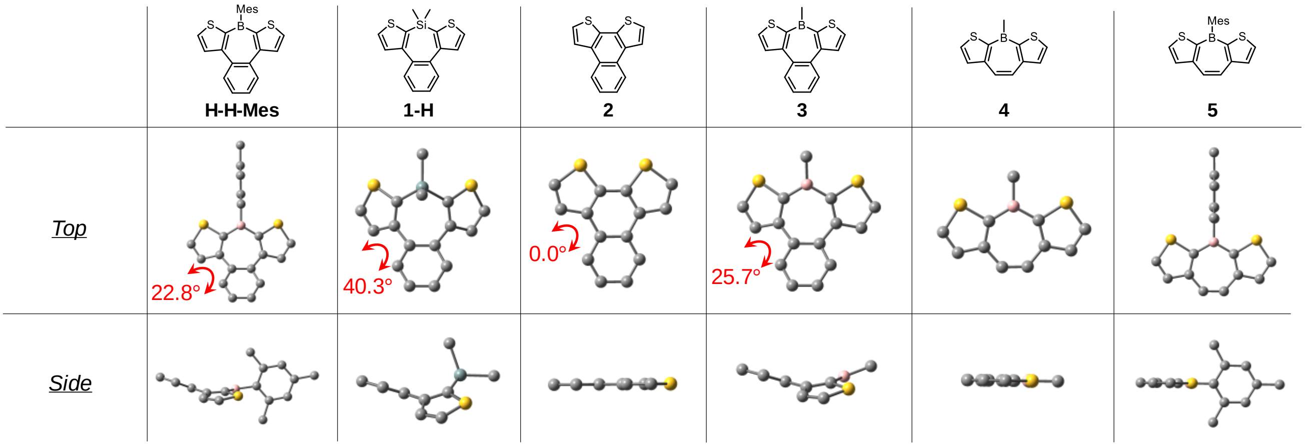

Figure S14 Optimized structures of model compounds in S_0 geometry at the B3LYP/6-31G(d) level of theory.

Table S5 TD-DFT results of model compounds at the S_0 geometries at the B3LYP/6-31G(d) level.

Compound	Excited State	Composition	CI	Excitation energy /eV	Wavelength /nm	Oscillator strength
**H-H-Mes**	S0→S1	H→L	0.70175	3.35	370	0
	S0→S2	H-2→L	0.57466	3.58	346	0.01
		H-1→L	0.39831			
	S0→S3	H-2→L	-0.39242	3.63	341	0.1
		H-1→L	0.54303			
	S0→S4	H-3→L	0.67166	3.76	330	0.07
	S0→S5	H-4→L	0.67951	4.13	301	0.04
	S0→T1	H-3→L	0.59001	2.73	455	0
		H-1→L+1	0.2487			

	S0→T2	H-2→L	-0.21623	2.93	423	0
		H-1→L	0.60002			
	S0→T3	H-4→L	-0.29018	3.29	377	0
		H-1→L+1	0.26835			
		H→L	0.53218			
	S0→T4	H-4→L	0.30934	3.37	368	0
		H-1→L+1	-0.30658			
		H→L	0.43825			
	S0→T5	H-5→L	0.41454	3.49	356	0
		H-3→L+1	-0.38815			
		H-1→L	0.24218			
**1-H**	S0→S1	H-1→L	-0.45213	3.1	399	0
		H→L+1	0.46756			
	S0→S2	H-1→L+1	0.43198	3.27	379	0
		H→L	-0.40458			
	S0→S3	H-2→L	0.27097	3.63	342	0
		H-1→L	0.2332			
		H-1→L+2	0.29103			
		H→L+1	0.2548			
	S0→S4	H-3→L	-0.35958	4.06	305	0
		H-2→L+1	0.27729			
		H→L	0.43288			
	S0→S5	H-1→L	0.32687	4.13	300	0
		H-1→L+2	-0.29265			
		H→L+1	0.4098			
**2**	S0→S1	H→L	0.68367	3.88	320	0.17
	S0→S2	H-1→L	0.58272	4.06	305	0.03
		H→L+1	0.38892			
	S0→S3	H-2→L	0.54373	4.58	271	0
		H→L+2	0.44608			
	S0→S4	H→L+3	0.69955	4.62	268	0
	S0→S5	H-1→L	-0.35929	4.89	254	0.36
		H→L+1	0.51989			
	S0→T1	H→L	0.67221	2.55	487	0
	S0→T2	H-2→L	-0.41595	3.46	358	0
		H→L+2	0.45215			
	S0→T3	H-1→L	0.68495	3.53	351	0
	S0→T4	H→L+1	0.56349	3.94	314	0
		H→L+4	0.26232			

	S0→T5	H-3→L	0.29946	3.99	311	0
		H→L+1	-0.38469			
		H→L+4	0.38823			
	S0→S1	H-1→L+1	0.45427	4.39	282	0
		H→L	0.52772			
	S0→S2	H→L+1	0.63637	4.58	271	0.03
	S0→S3	H-1→L	0.62034	4.7	264	0.11
		H→L+1	0.28131			
	S0→S4	H-1→L+1	-0.32811	4.96	250	0.18
		H→L	0.36342			
		H→L+2	0.43811			
	S0→S5	H-2→L+1	0.43094	4.98	249	0.08
		H-1→L+1	0.36917			
**3**	S0→S1	H→L	0.6684	3.7	335	0.08
	S0→S2	H-1→L	0.68025	3.81	326	0.08
	S0→S3	H-2→L	0.67665	4.23	293	0.04
	S0→S4	H-3→L	0.4897	4.54	273	0.06
		H-1→L+1	0.45662			
	S0→S5	H→L+1	0.65184	4.64	267	0.36
	S0→T1	H-1→L	0.60651	2.77	448	0
		H→L+1	0.27301			
	S0→T2	H-1→L+1	0.22306	2.98	417	0
		H→L	0.624			
	S0→T3	H-2→L	0.38152	3.38	367	0
		H→L+1	0.4485			
	S0→T4	H-3→L	0.41092	3.54	351	0
		H-1→L+1	-0.38274			
		H→L	0.29955			
	S0→T5	H-2→L	0.50497	3.74	332	0
		H-1→L	0.23759			
		H→L+1	-0.34278			
**4**	S0→S1	H→L	0.65804	3.76	330	0.06
	S0→S2	H-1→L	0.39648	4.02	308	0.03
		H→L+1	0.57599			
	S0→S3	H-2→L	0.2789	4.55	273	0.03
		H-1→L+1	0.59782			
	S0→S4	H-1→L	0.52842	4.66	266	0.65
		H→L+1	-0.36282			
	S0→S5	H-3→L	0.19538	5.08	244	0.01

		H-2→L	-0.38328			
		H-2→L+1	0.50786			
	S0→T1	H→L+1	0.63264	2.65	468	0
	S0→T2	H→L	0.67472	3	414	0
	S0→T3	H-2→L+2	0.15757	3.37	368	0
		H-1→L	0.61114			
	S0→T4	H-2→L	-0.3216	3.49	355	0
		H-1→L+1	0.56509			
	S0→T5	H-2→L	0.50912	4.08	304	0
		H-1→L+1	0.37351			
**5**	S0→S1	H-1→L	0.70483	3.57	347	0
	S0→S2	H→L	0.66076	3.72	333	0.07
	S0→S3	H-2→L	0.70597	3.8	326	0
	S0→S4	H-1→L+1	0.70575	3.85	322	0
	S0→S5	H-3→L	0.40897	3.98	312	0.02
		H→L+1	0.56778			
	S0→T1	H-3→L	0.22442	2.66	467	0
		H→L+1	0.63188			
	S0→T2	H→L	0.68084	2.97	418	0
	S0→T3	H-3→L	0.61503	3.33	372	0
		H→L+1	-0.26273			
	S0→T4	H-4→L	-0.30596	3.48	356	0
		H-3→L+1	0.58149			
	S0→T5	H-1→L	0.70395	3.55	350	0

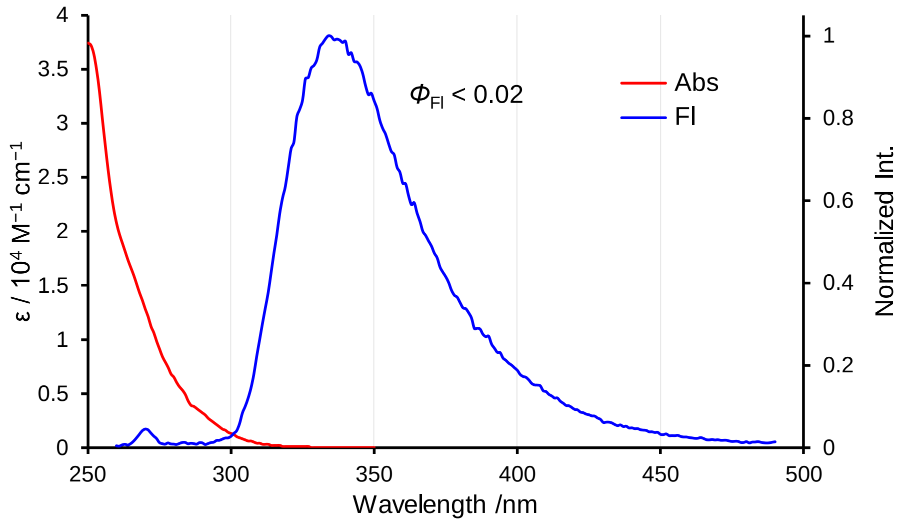

Figure S15 Absorption and fluorescence spectra of **1-H** in 2-MeTHF at room temperature.

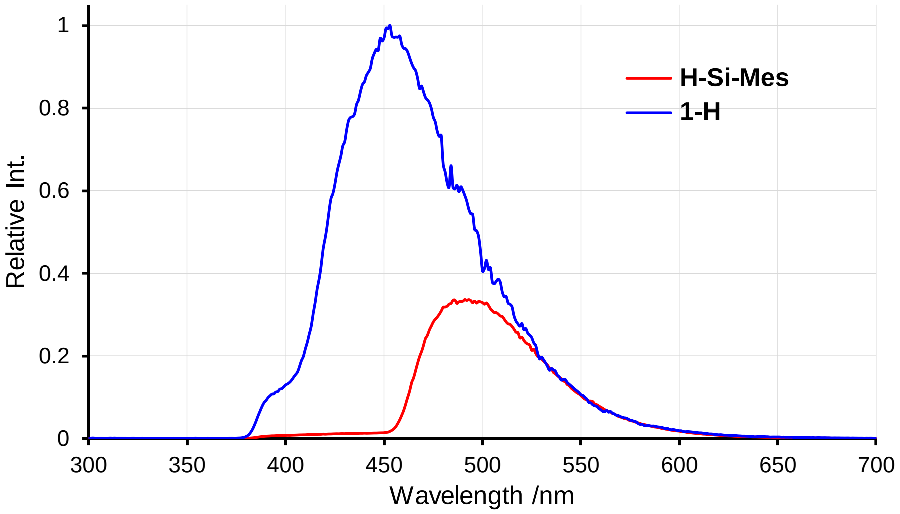

Figure S16 Relative phosphorescence spectra of **H-Si-Mes** and **1-H** in 2-MeTHF at 77 K. The intensity is corrected by the absorption coefficients in 2-MeTHF.

Table S6 TD-DFT results of benzo[*d*]dithieno[*b*,*f*]borepins in T_1 geometry at the B3LYP/6-31G(d) level.

Compound	Excited State	Composition	CI	Excitation energy /eV	Wavelength /nm	Oscillator strength
**H-Si-Mes**	S0→S1	H-1→L	0.532	3.00	413	0.05
		H→L	-0.459			
	S0→S2	H-1→L	0.458	3.16	393	0.14
		H→L	0.515			
	S0→T1	H→L	0.649	1.96	632	0.00
	S0→T2	H-3→L	0.618	2.71	458	0.00
	S0→T3	H-1→L	0.67	2.99	414	0.00
	S0→T4	H-5→L	0.451	3.16	392	0.00
		H-3→L+1	0.319			
	S0→T5	H-4→L	-0.331	3.23	384	0.00
		H-2→L	0.343			
		H→L+1	0.318			
**OMe-H-Mes**	S0→S1	H→L	0.685	2.97	418	0.21
	S0→S2	H-1→L	0.696	3.25	381	0.00
	S0→S3	H-2→L	0.645	3.44	360	0.03
	S0→S4	H-3→L	0.688	3.48	357	0.00
	S0→S5	H-4→L	0.557	3.93	316	0.01
		H→L+1	-0.402			
		H→L+1	-0.402			
	S0→T1	H→L	0.635	2.03	611	0.00

	S0→T2	H-2→L	0.562	2.75	451	0.00
		H→L+1	-0.132			
	S0→T3	H→L+1	0.597	3.06	405	0.00
	S0→T4	H-1→L	0.647	3.21	386	0.00
	S0→T5	H-6→L	0.318	3.32	374	0.00
		H-5→L	-0.174			
		H-2→L+1	0.412			
**F-H-^FMes**	S0→S1	H→L	0.681	3.14	395	0.15
	S0→S2	H-1→L	0.664	3.42	363	0.07
	S0→S3	H-2→L	0.619	3.85	322	0.02
	S0→S4	H→L+1	0.618	3.88	319	0.01
	S0→S5	H→L+2	0.675	4.08	304	0.00
	S0→T1	H→L	0.651	1.96	632	0.00
	S0→T2	H-1→L	0.639	2.77	447	0.00
	S0→T3	H-3→L	0.32	3.21	386	0.00
		H-2→L	0.312			
		H-1→L+3	0.341			
		H→L+3	0.244			
	S0→T4	H-3→L	-0.301	3.27	379	0.00
		H-2→L	0.222			
		H-1→L+3	-0.219			
		H→L+3	0.41			
	S0→T5	H-2→L	0.485	3.46	358	0.00
		H-1→L+3	-0.184			
		H→L+3	-0.286			

Table S7 SOCMEs of benzo[*d*]dithieno[*b*,*f*]borepins in cm^{-1} at the T_1 geometries at the B3LYP/6-31G(d) level.

SOCME		**H-Si-Mes**	**OMe-H-Mes**	**F-H-^{F}Mes**	**H-H-Mes**
T_n	S_n				
1	0	10.58	9.08	12.15	10.69
1	1	2.76	1.81	2.57	2.59
2	1	4.87	2.25	2.54	4.85
3	1	2.83	3.55	0.39	2.2
4	1	4.64	6.22	6.15	3.88
5	1	5.45	1.91	5.72	1.66

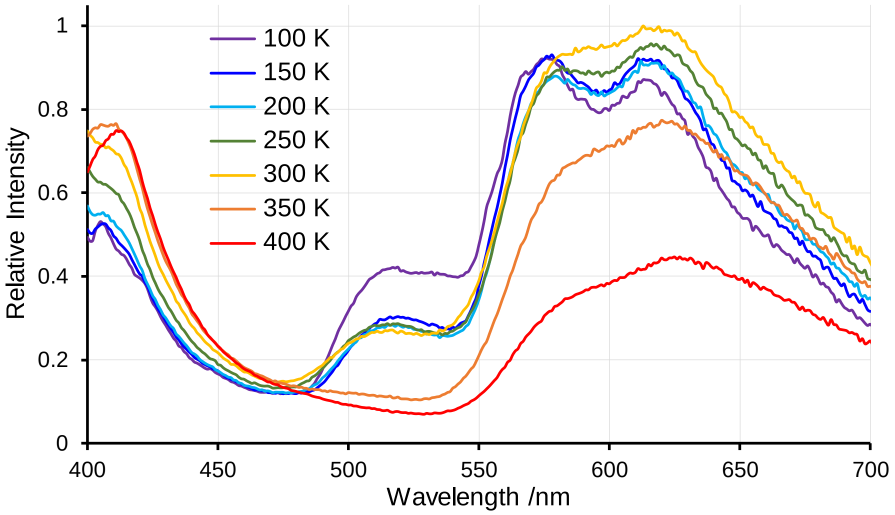

Figure S17 VT photoluminescence spectra of **H-Si-Mes**_C^R from 100 to 400 K in air.

Table S8 Emission lifetime of **H-Si-Mes**_C^R at various wavelengths under 100 K.

Wavelength / nm	τ (ratio) / ms
520	69.4 (11) / 182 (89)
620	138 (30) / 39.8 (70)

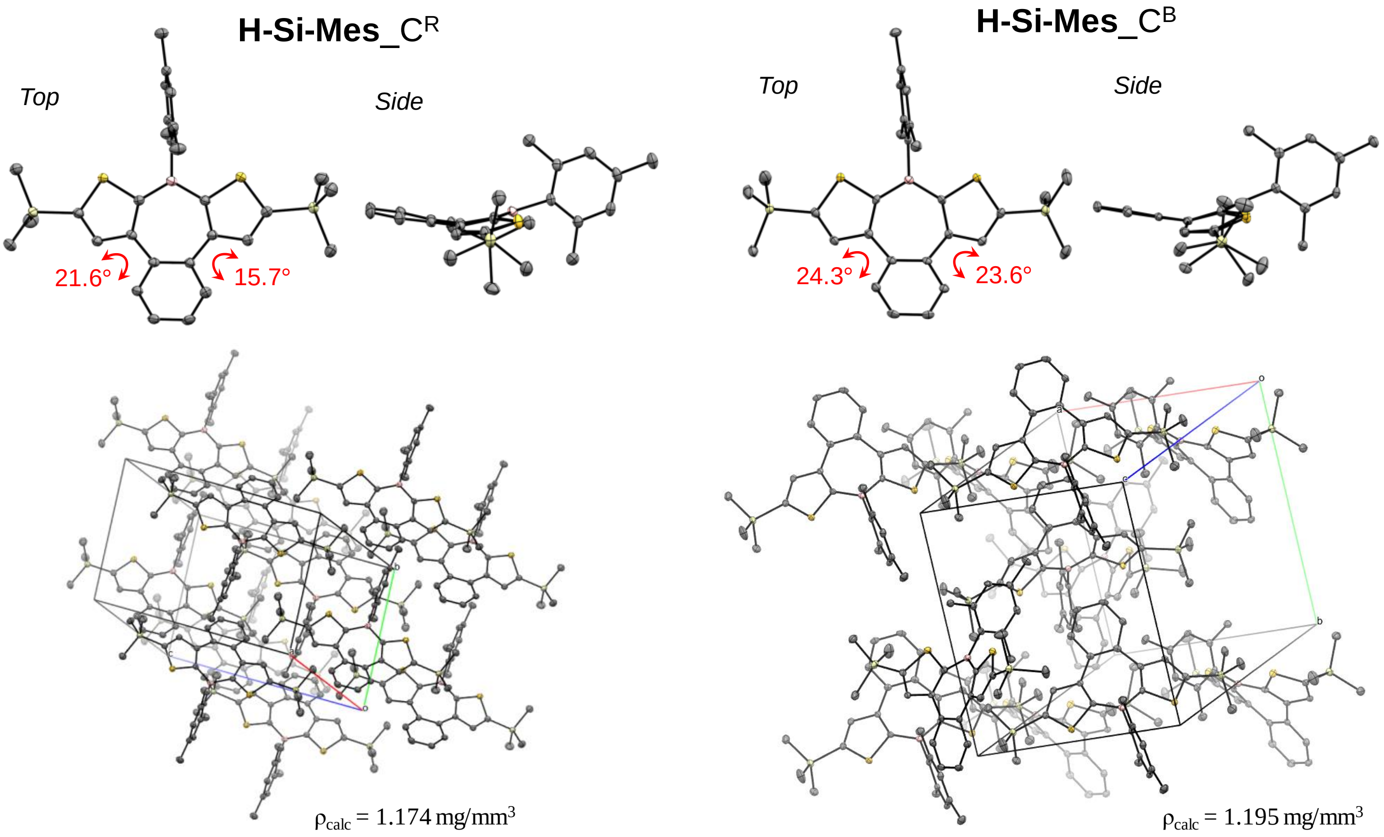

Figure S18 ORTEP drawings and packing diagrams of **H-Si-Mes**.

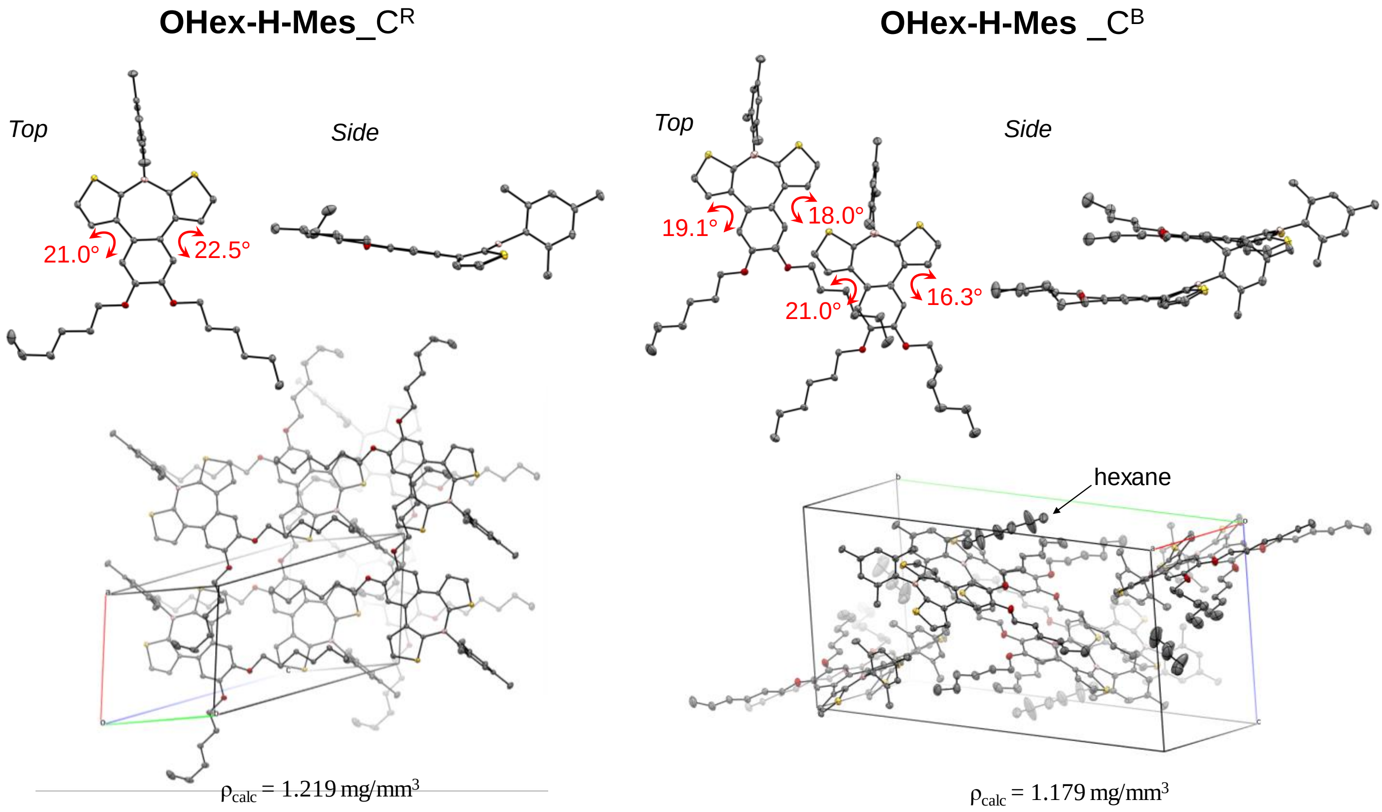

Figure S19 ORTEP drawings and packing diagrams of **OHex-H-Mes**.

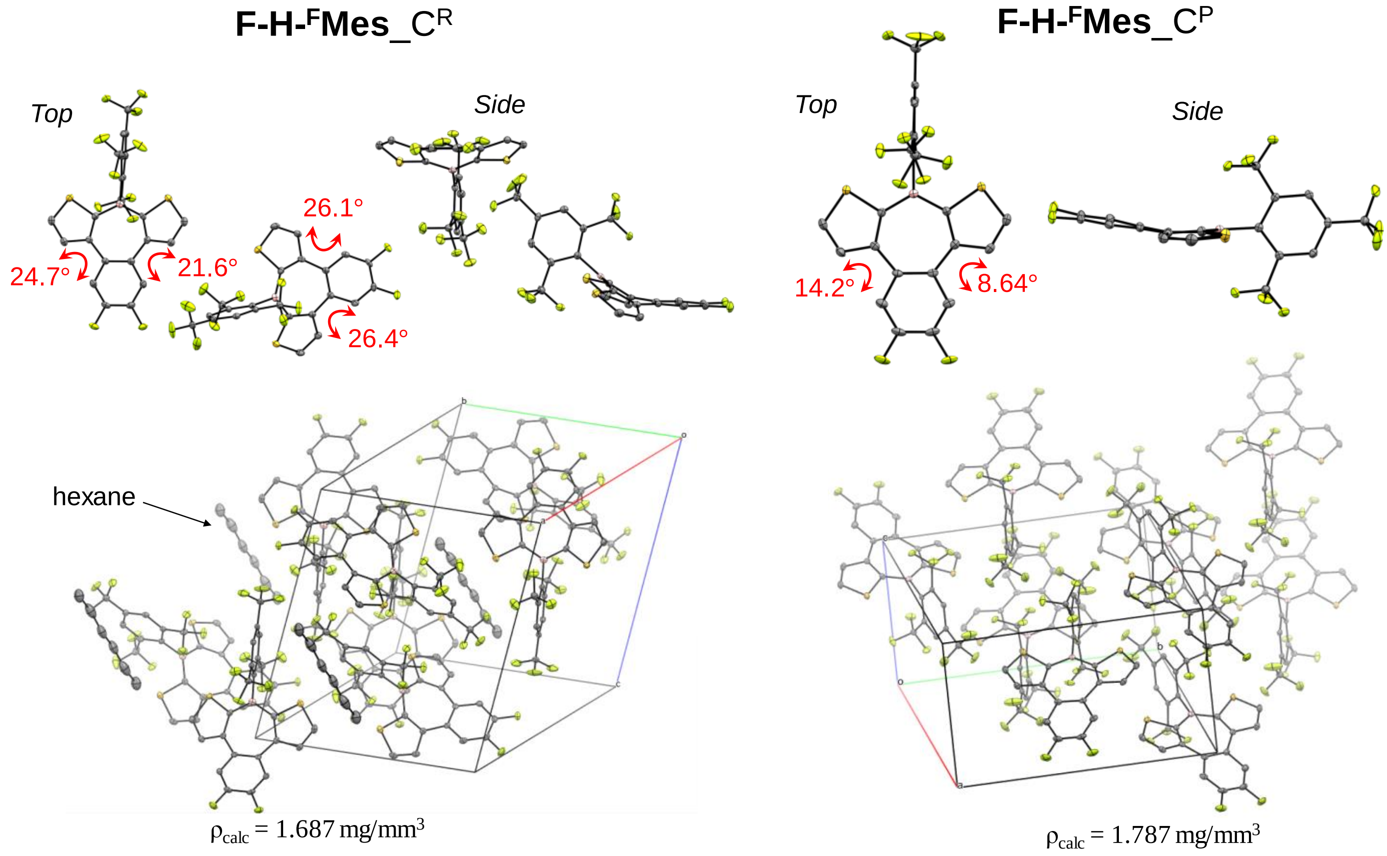

Figure S20 ORTEP drawings and packing diagrams of **F-H-^{F}Mes**.

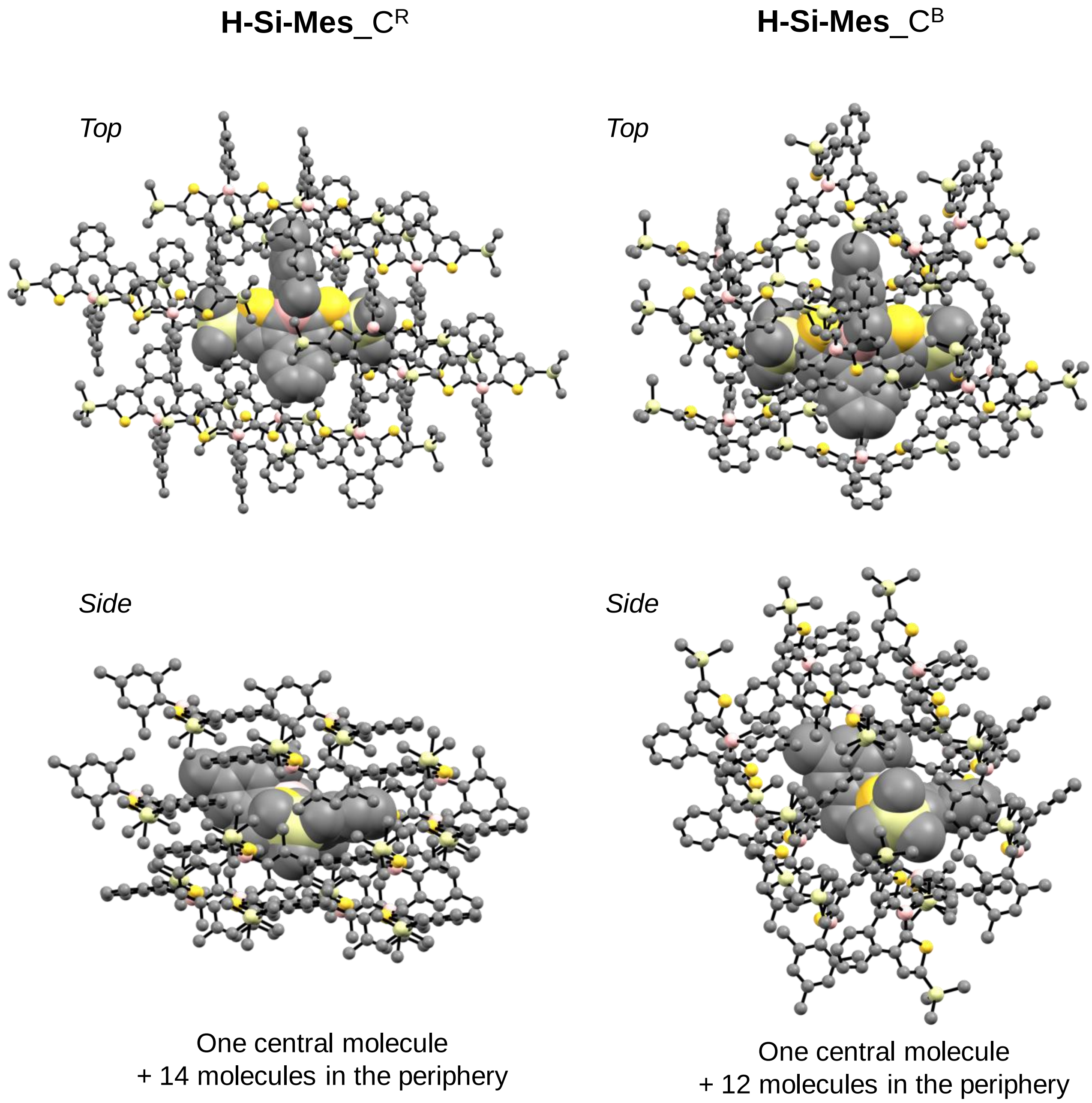

Figure S21 Computational model of NEDA calculations for **H-Si-Mes**. The central and surrounding molecules are shown in the spacefill and ball and stick models, respectively.

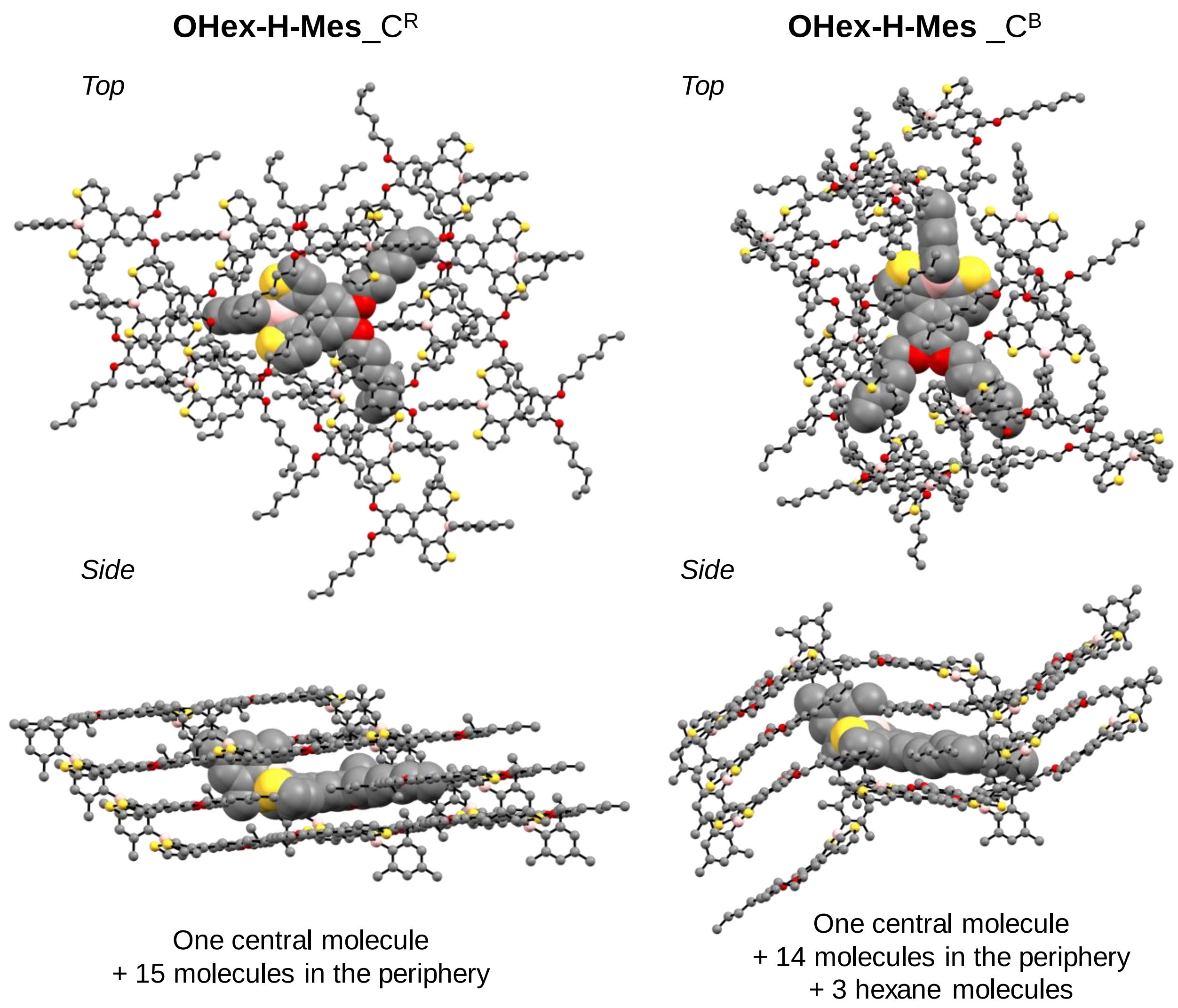

Figure S22 Computational model of NEDA calculations for **OHex-H-Mes**. The central and surrounding molecules are shown in the spacefill and ball and stick models, respectively.

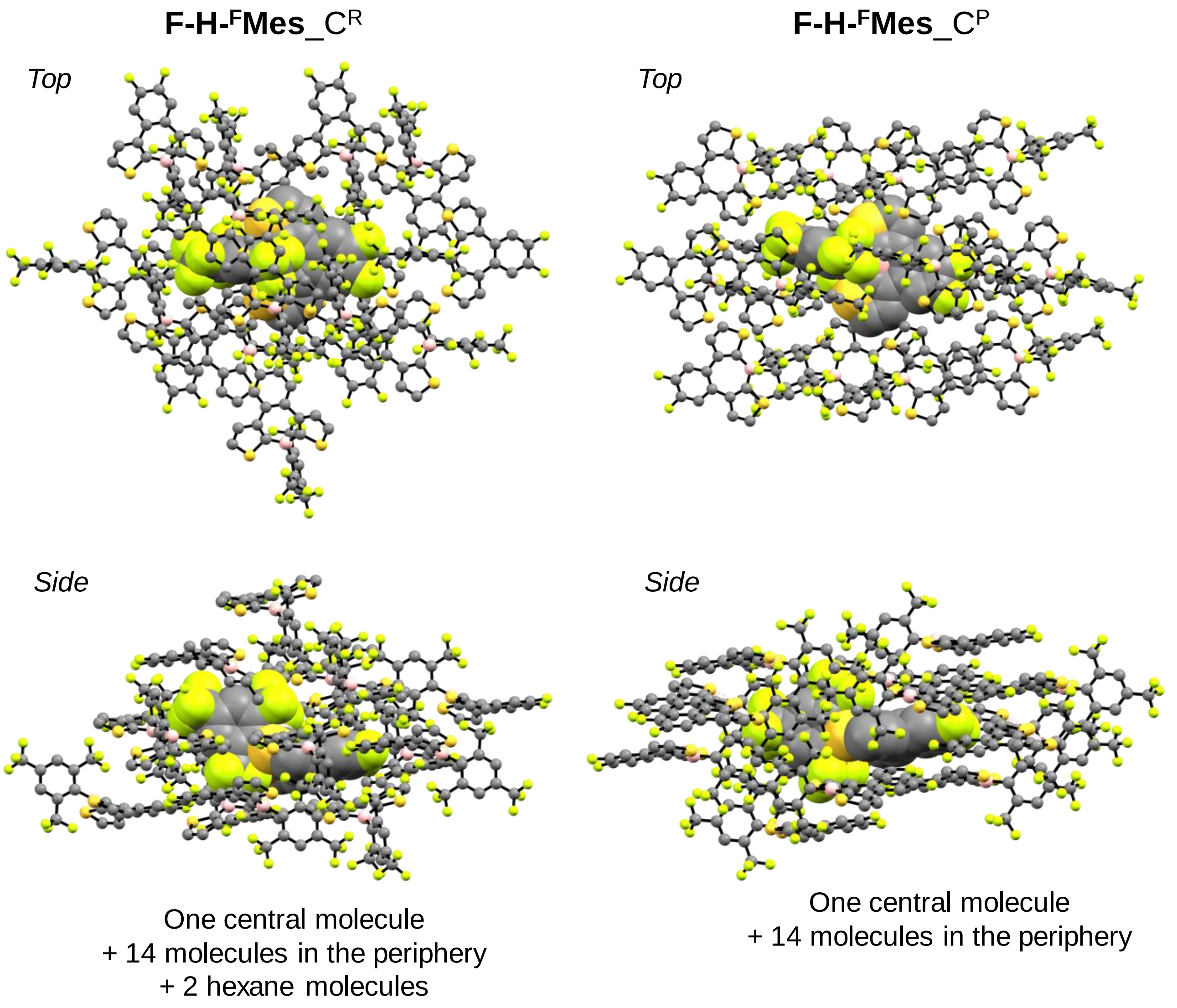

Figure S23 Computational model of NEDA calculations for **F-H-^{F}Mes**. The central and surrounding molecules are shown in the spacefill and ball and stick models, respectively.

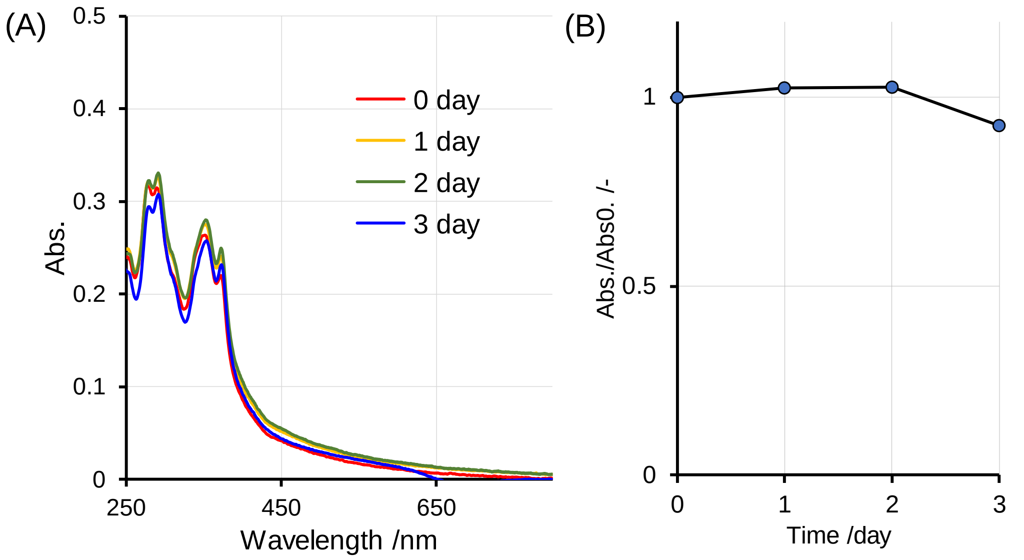

Figure S24 Absorption spectra (A) and absorbance changes at 343 nm (B) of **H-Si-Mes** complexed with P123 in aqueous media.

Table S9 Solution properties of complex of **H-Si-Mes** with P123.

	D_{hy} /nm[a]	PDI[a]	ζ-potential /mV[b]	[**H-Si-Mes**] /µM[c]
**H-Si-Mes**/P123	190 ±6	0.286	-9.0 ±0.5	63.6

[a] Hydrodynamic diameter (D_{hy}) were determined by DLS and PDI was calculated by cumulant method. [b] The samples were measured in MilliQ (pH, 7.4; 25 °C). [c] Concentration of **H-Si-Mes** was determined by absorbance.

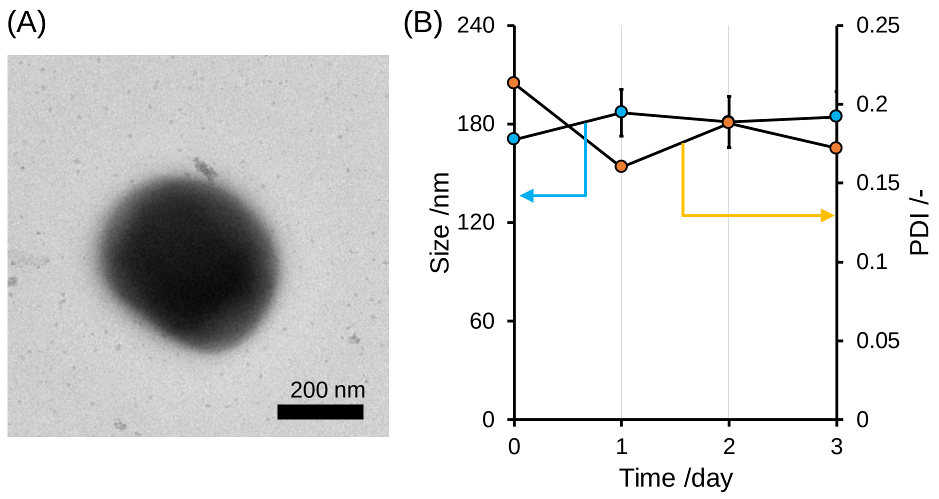

Figure S25 (A) Representative morphology of **H-Si-Mes** with P123 observed by transmission electron microscopy. The samples were stained with 0.1 % ammonium molybdate. (B) Change in particle size and PDI.

Hydrophobicity test of micelles

We examined the encapsulation mechanism using P123 by pyrene since the ratio of fluorescence intensities at 375 (I_1) and 385 nm (I_2) work as the indicator for polarity in microenvironment such as hydrophobic core in micelles[S3] and cross-linking point in amphiphilic self-assembled nanogels.[S4] When pyrenes were dispersed with P123, the ratio was determined to be 0.66 (Fig. S25), which is relatively small and indicates that hydrophobic compounds can be trapped in hydrophobic nanodomain in P123 micelles.

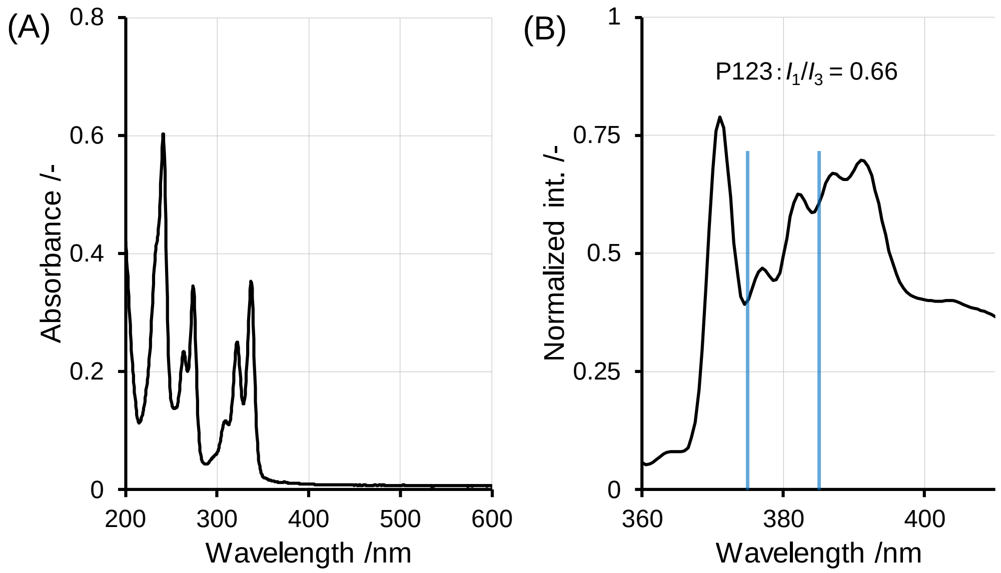

Figure S26 Absorption (A) and fluorescence spectra (B) of pyrene complexed with P123 in aqueous media.

References

[S1] Y. Adachi and J. Ohshita, *Organometallics* **2018**, *37*, 869−881.

[S2] Y. Adachi, F. Arai, and F. Jäkle, *Chem. Commun.* **2020**, *56*, 5119−5122.

[S3] L. Piñeiro, M. Novo, W. Al-Soufi, *Adv. Colloid Interface Sci.* **2015**, *215*, 1−12.

[S4] R. Kawasaki, Y. Sasaki, K. Akiyoshi, *Chem. Lett.* **2017**, *46*, 513−515.

NMR spectra

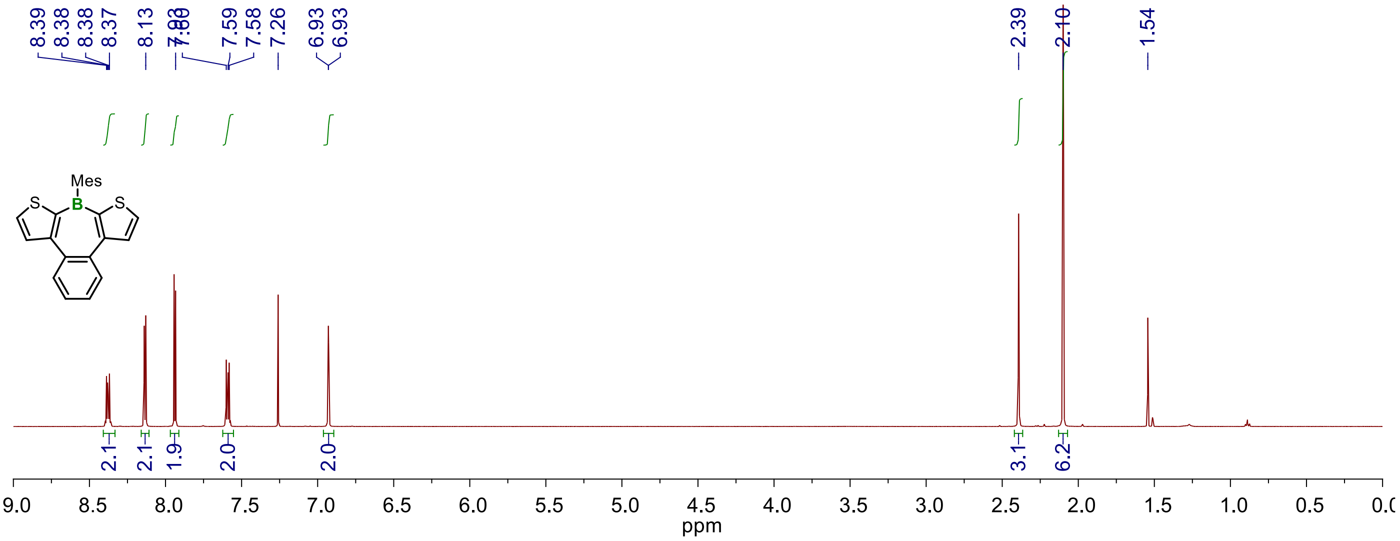

Figure S27 ^{1}H NMR spectrum of **H-H-Mes** in $CDCl_3$ at room temperature (500 MHz).

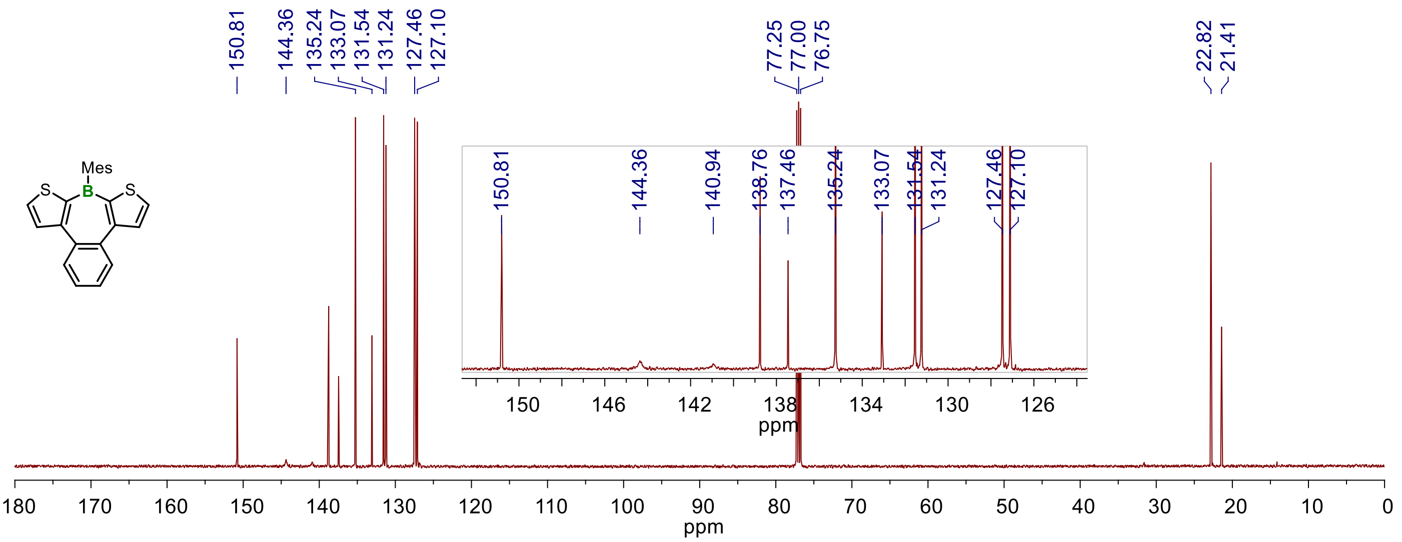

Figure S28 ^{13}C NMR spectrum of **H-H-Mes** in $CDCl_3$ at room temperature (126 MHz).

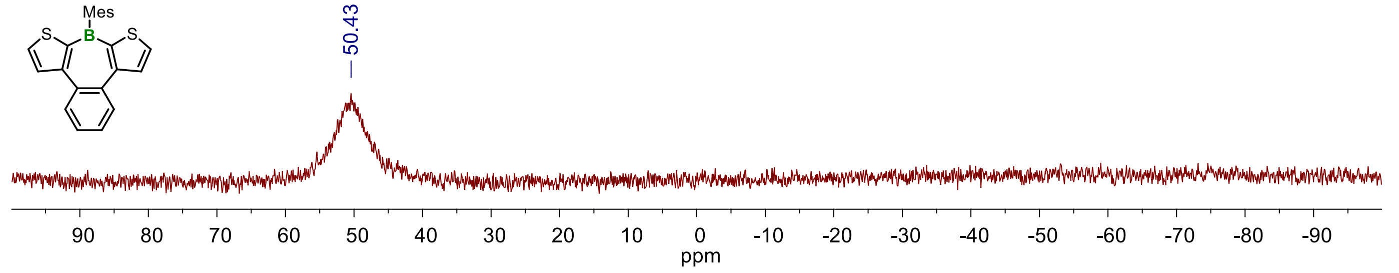

Figure S29 ^{11}B NMR spectrum of **H-H-Mes** in $CDCl_3$ at room temperature (160 MHz).

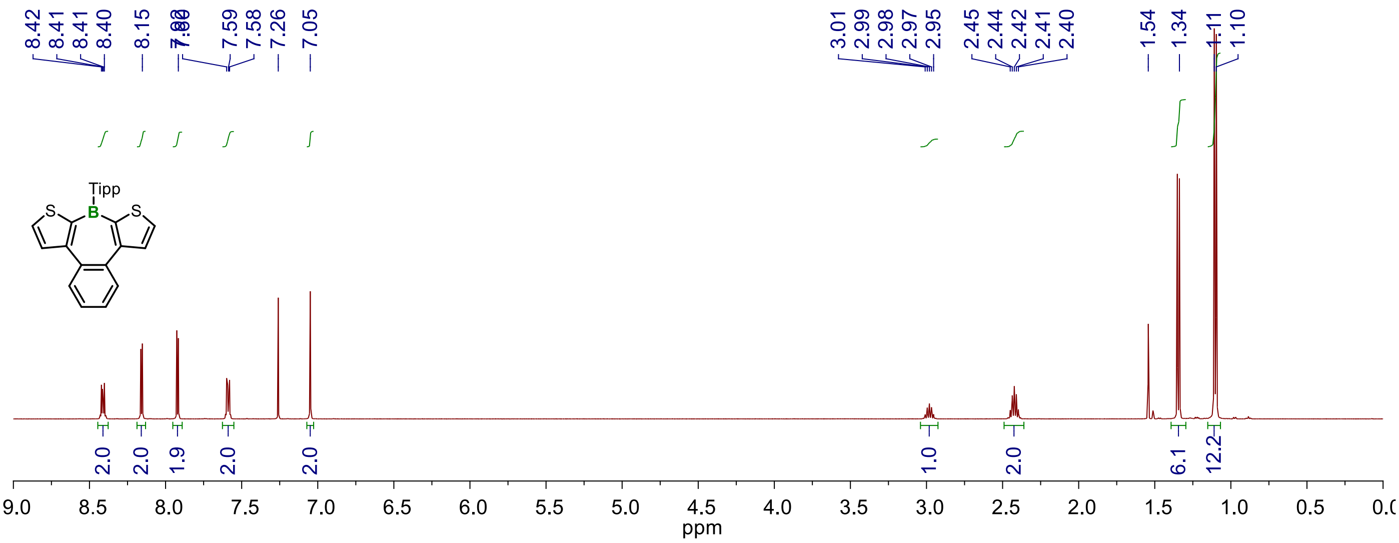

Figure S30 ^{1}H NMR spectrum of **H-H-Tipp** in $CDCl_3$ at room temperature (500 MHz).

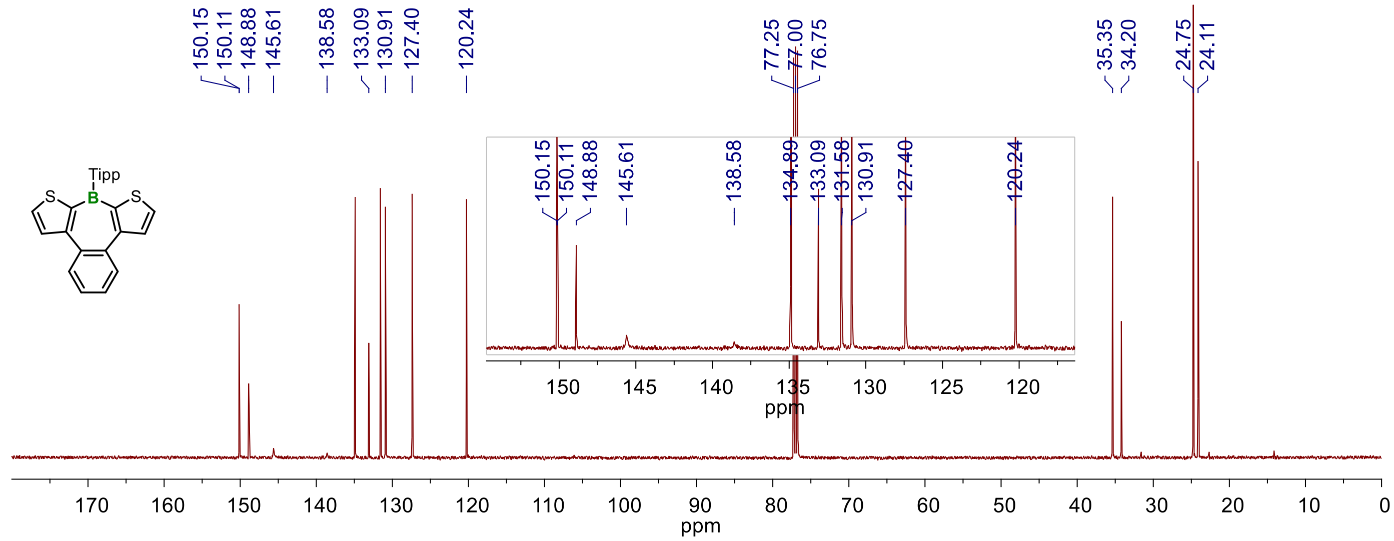

Figure S31 ^{13}C NMR spectrum of **H-H-Tipp** in $CDCl_3$ at room temperature (126 MHz).

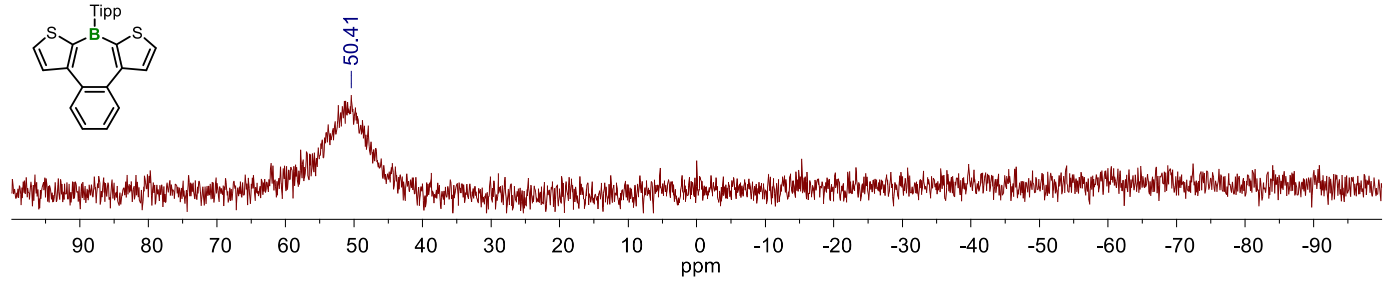

Figure S32 ^{11}B NMR spectrum of **H-H-Tipp** in $CDCl_3$ at room temperature (160 MHz).

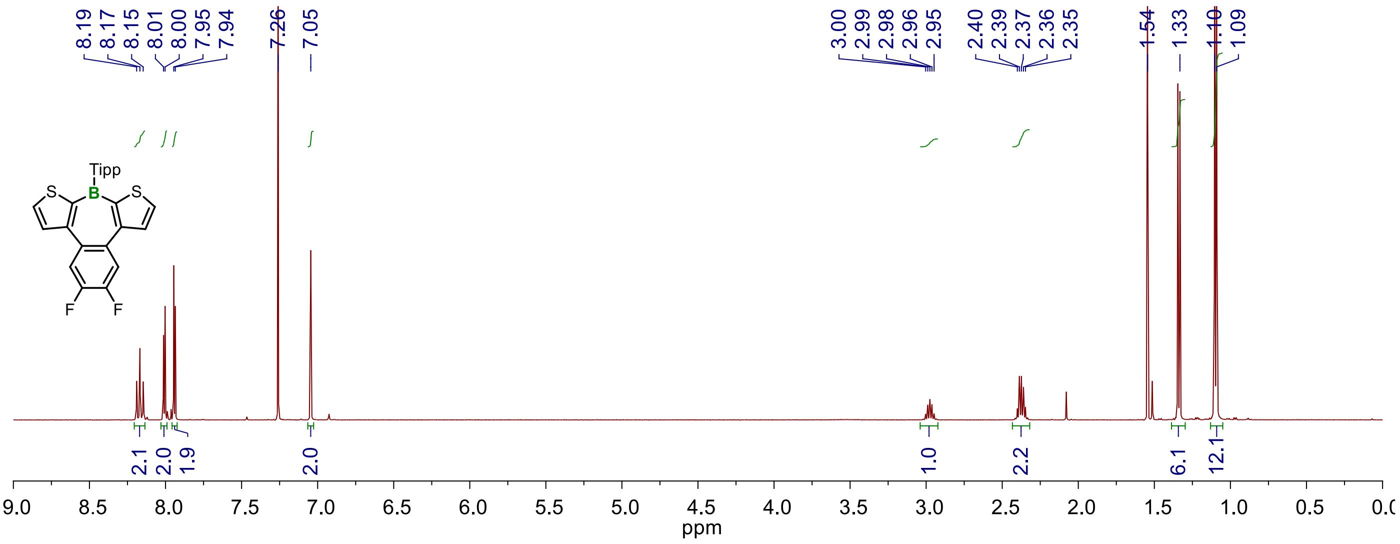

Figure S33 ^{1}H NMR spectrum of **F-H-Tipp** in $CDCl_3$ at room temperature (500 MHz).

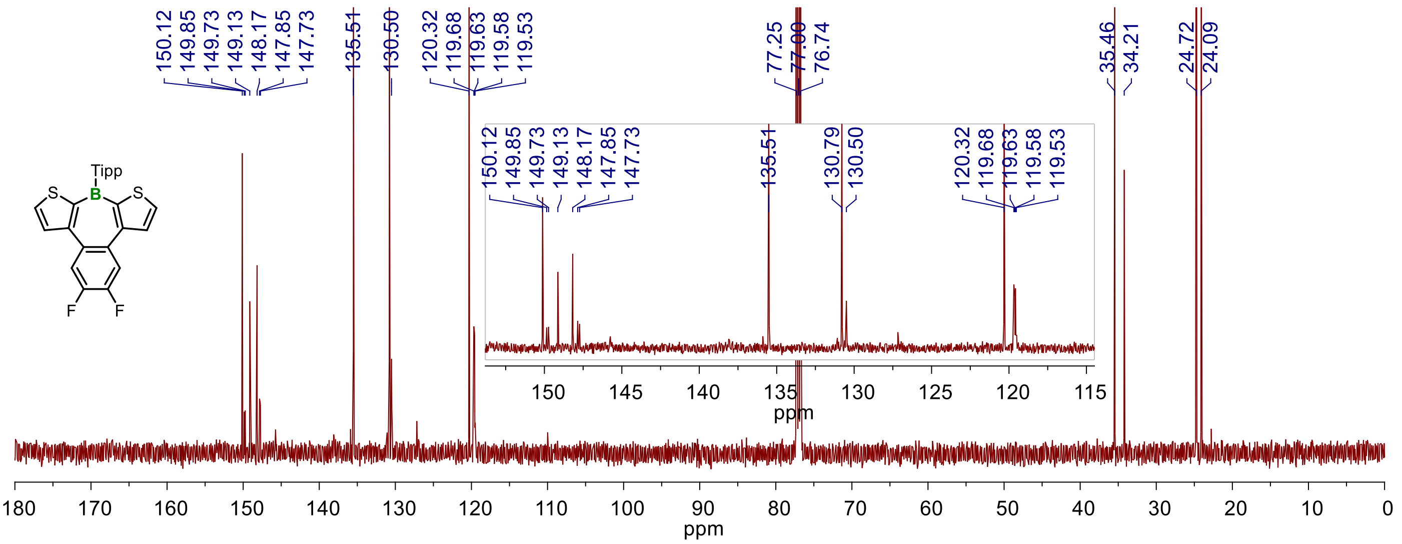

Figure S34 ^{13}C NMR spectrum of **F-H-Tipp** in $CDCl_3$ at room temperature (126 MHz).

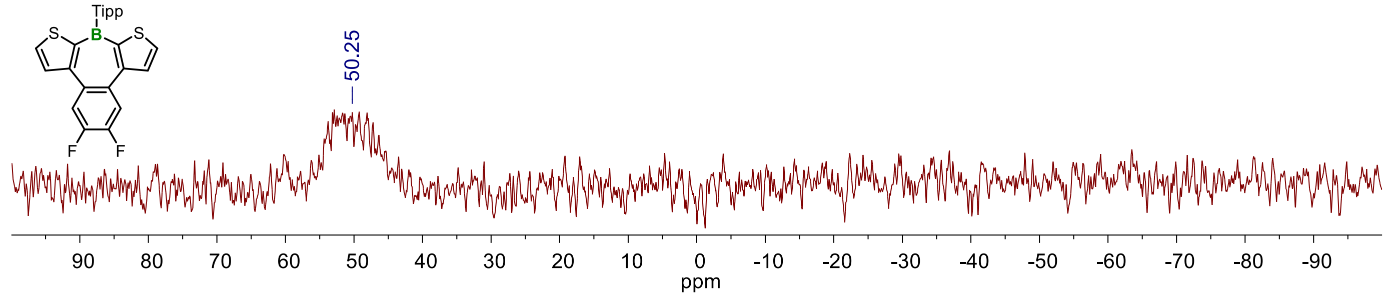

Figure S35 ^{11}B NMR spectrum of **F-H-Tipp** in $CDCl_3$ at room temperature (160 MHz).

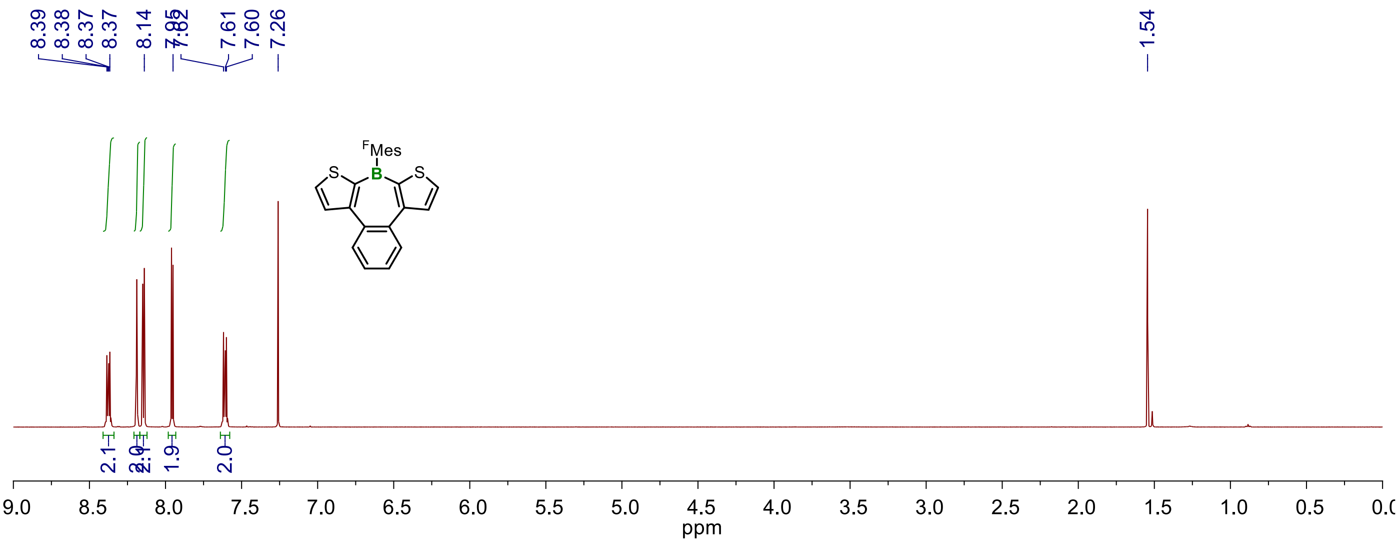

Figure S36 ^{1}H NMR spectrum of **H-H-^{F}Mes** in $CDCl_3$ at room temperature (500 MHz).

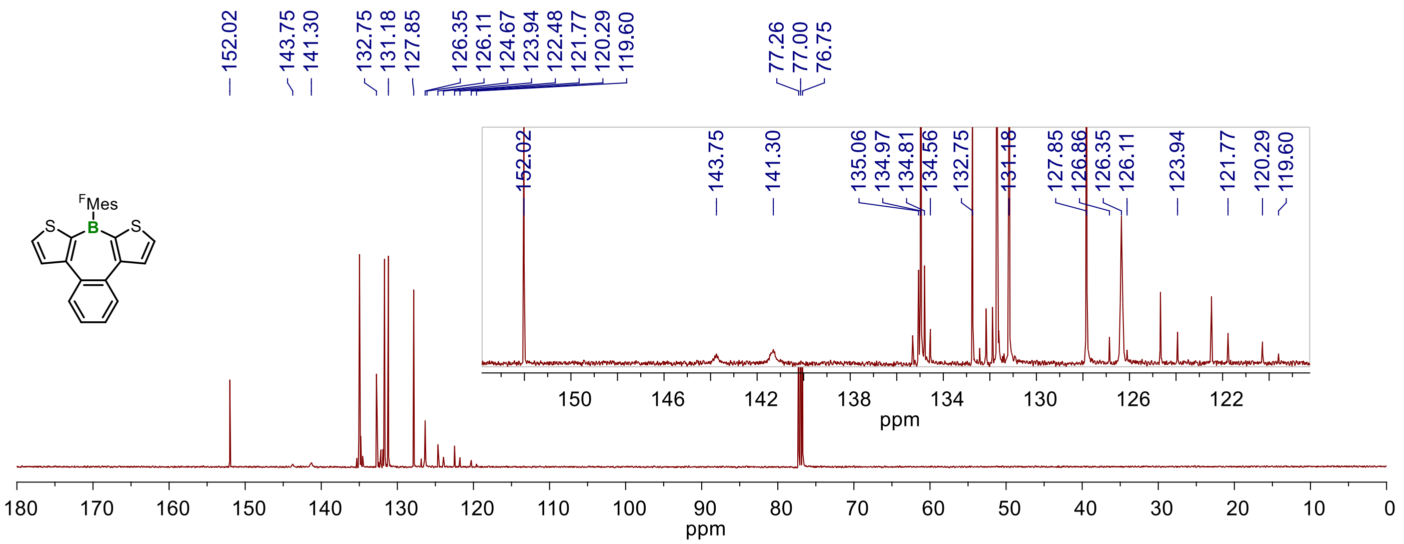

Figure S37 ^{13}C NMR spectrum of **H-H-^{F}Mes** in $CDCl_3$ at room temperature (126 MHz).

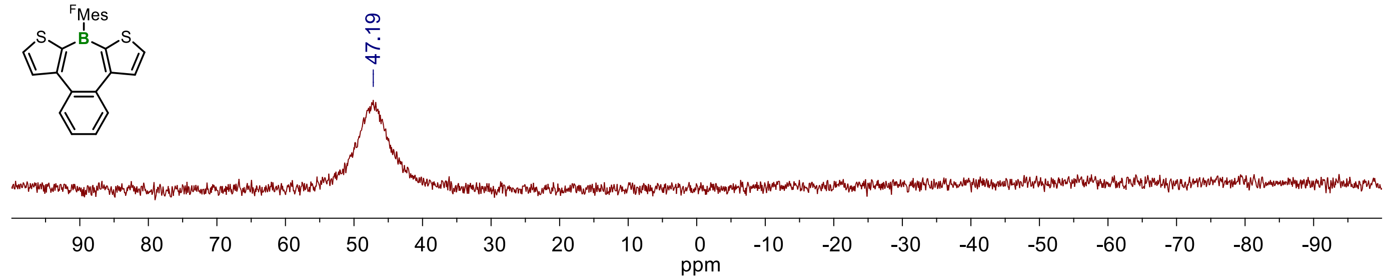

Figure S38 ^{11}B NMR spectrum of **H-H-^{F}Mes** in $CDCl_3$ at room temperature (160 MHz).

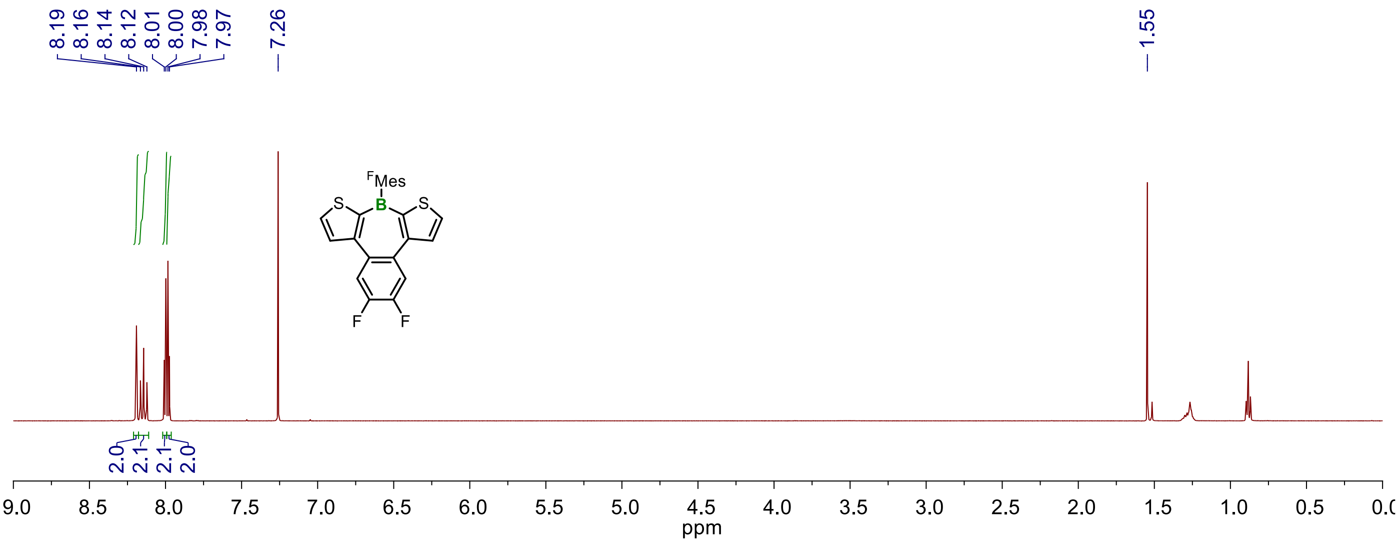

Figure S39 ^{1}H NMR spectrum of **F-H-^{F}Mes** in $CDCl_3$ at room temperature (500 MHz).

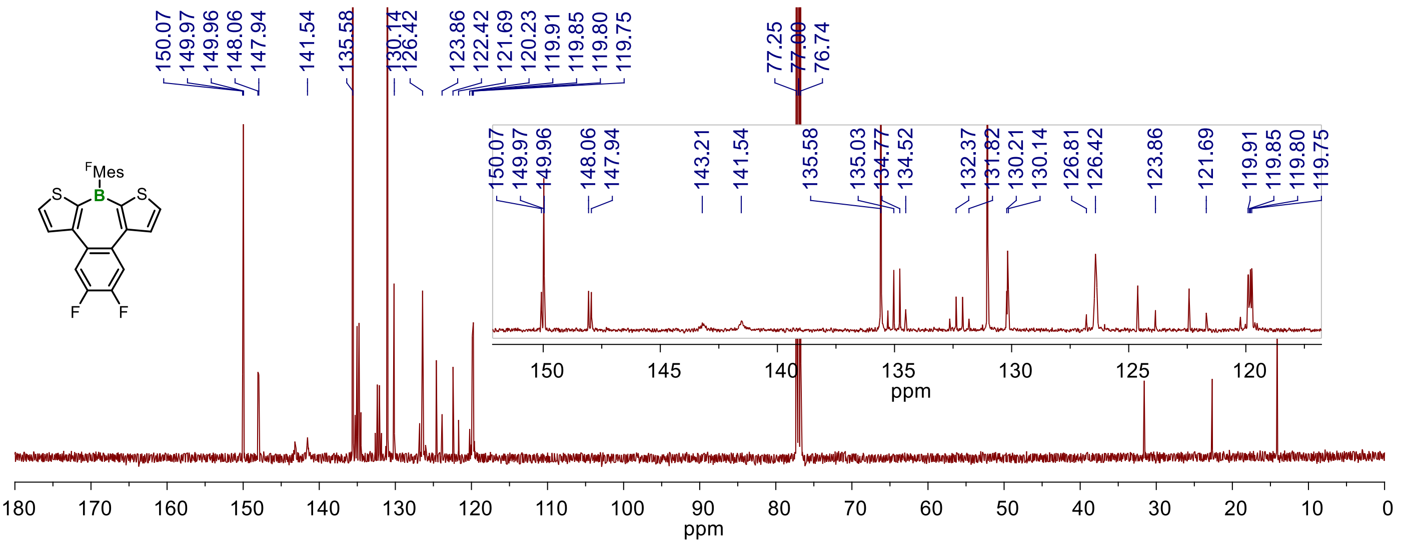

Figure S40 ^{13}C NMR spectrum of **F-H-^{F}Mes** in $CDCl_3$ at room temperature (126 MHz).

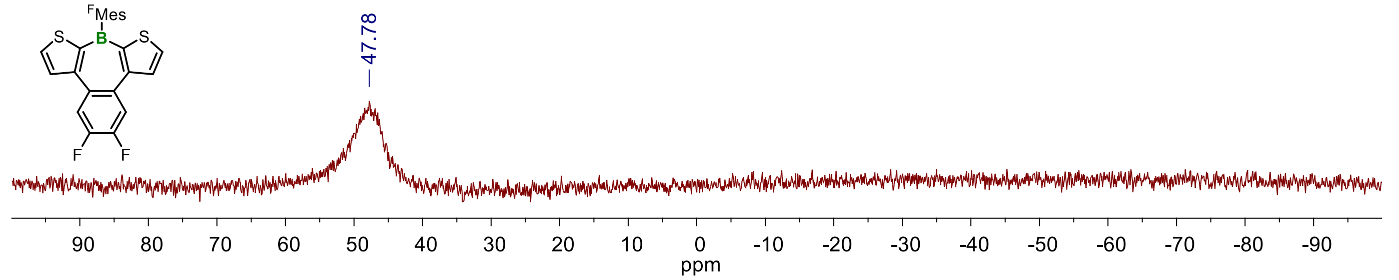

Figure S41 ^{11}B NMR spectrum of **F-H-^{F}Mes** in $CDCl_3$ at room temperature (160 MHz).
